# Stanniocalcin 1 promotes metastasis, lipid metabolism and cisplatin chemoresistance via the FOXC2/ITGB6 signaling axis in ovarian cancer

**DOI:** 10.1186/s13046-022-02315-3

**Published:** 2022-04-07

**Authors:** Feikai Lin, Xiaoduan Li, Xinjing Wang, Huizhen Sun, Ziliang Wang, Xipeng Wang

**Affiliations:** 1 Department of Gynecology and Obstetrics, Xinhua Hospital Affiliated to Shanghai Jiaotong University School of Medicine, Shanghai, 200092 People’s Republic of China; 2 Department of Gynecology, Shanghai First Maternity and Infant Hospital, Tongji University School of Medicine, Shanghai, 201204 People’s Republic of China

**Keywords:** STC1, Metastasis, Lipid metabolism, DDP chemoresistance, OC

## Abstract

**Background:**

Stanniocalcin 1 (STC1) plays an integral role in ovarian cancer (OC). However, the functional role of STC1 in metastasis, lipid metabolism and cisplatin (DDP) chemoresistance in OC is not fully understood.

**Methods:**

Single-cell sequencing and IHC analysis were performed to reveal STC1 expression profiles in patient tissues. Metastasis, lipid metabolism and DDP chemoresistance were subsequently assessed. Cell-based in vitro and in vivo assays were subsequently conducted to gain insight into the underlying mechanism of STC1 in OC.

**Results:**

Single-cell sequencing assays and IHC analysis verified that STC1 expression was significantly enhanced in OC tissues compared with para-carcinoma tissues, and it was further up-regulated in peritoneal metastasis tissues compared with OC tissues. In vitro and in vivo experiments demonstrated that STC1 promoted metastasis, lipid metabolism and DDP chemoresistance in OC. Simultaneously, STC1 promoted lipid metabolism by up-regulating lipid-related genes such as UCP1, TOM20 and perilipin1. Mechanistically, STC1 directly bound to integrin β6 (ITGB6) to activate the PI3K signaling pathway. Moreover, STC1 was directly regulated by Forkhead box C2 (FOXC2) in OC. Notably, targeting STC1 and the FOXC2/ITGB6 signaling axis was related to DDP chemoresistance in vitro.

**Conclusions:**

Overall, these findings revealed that STC1 promoted metastasis, lipid metabolism and DDP chemoresistance via the FOXC2/ITGB6 signaling axis in OC. Thus, STC1 may be used as a prognostic indicator in patients with metastatic OC. Meanwhile, STC1 could be a therapeutic target in OC patients, especially those who have developed chemoresistance to DDP.

**Supplementary Information:**

The online version contains supplementary material available at 10.1186/s13046-022-02315-3.

## Background

Ovarian cancer (OC) is the second most lethal gynecologic malignancy and the eighth most common cause of cancer-related death in women globally [1]. The prevalence of OC has increased in the past ten years [2]. Due to the lack of an effective early-stage screening method, more than 75% of OC patients have developed advanced-stage disease at the time of diagnosis [3]. The most effective treatment for advanced OC involves a surgery followed by six cycles of chemotherapy, while DDP is a platinum-based first-line drug for treating OC [4]. However, DDP chemoresistance is an important factor in the high mortality rates of OC. Therefore, accumulating studies have suggested that the treatment strategies for OC patients have to be designed from a patient's perspective and incorporate meaningful and beneficial measures [5]. Thus, it is important to identify novel prognostic biomarkers as well as potential therapeutic targets.

Accumulating evidence has revealed that lipid metabolic pathways are closely correlated with the poor prognosis of OC, as they provide the energy needed for cancer cells to rapidly proliferate and metastasize [6, 7]. Cancer metastasis depends on the complex interaction between cancer cells and the microenvironments in an organ [8–10]. Intra-abdominal tumors, including OC, have a clear predilection for metastasis to the omentum and some peritoneal tissues [11, 12], which are primarily composed of adipocytes and function as storage sites for energy-dense lipids [13]. Adipocytes are a major component of the omentum and provide fatty acids for rapid tumor growth, thus identifying lipid metabolism and transport as new targets for the treatment of OC [6]. On the other hand, cytoreductive surgery and combination platinum-taxane chemotherapy have remained the mainstay of OC therapy for decades [14, 15]. As DDP is a first-line chemotherapeutic drug for relapsed platinum-sensitive OC[16], many studies focused on the chemotherapeutic effect of DDP recently [17, 18]. However, the underlying mechanisms of metastasis, lipid metabolism and DDP chemoresistance in OC cells are still unclear.

The stanniocalcin (STC) family consists of two proteins, STC1 and STC2, which were initially identified in teleost fish as regulators of calcium homeostasis[19]; their human counterparts were first described in 1995 [13, 19]. STC1 is a secreted glycoprotein hormone that has been detected in various human tissues [20], such as spleen [21], kidney [22] and skeletal muscle[23] tissue. In addition, enhanced expression of STC1 has been identified in various cancers, such as breast cancer [24], hepatocellular carcinoma [25] and glioblastoma [26]. In the context of female reproductive endocrinology, STC1 was determined to be overexpressed in OC [27] and to modulate ovarian function [28], implantation [29] and other processes [30, 31]. It has been reported that STC1 is involved in OC cell proliferation and migration [27, 32]. However, further studies are needed to elucidate the regulatory mechanisms of STC1 in metastasis, lipid metabolism and DDP chemoresistance in OC to guide personalized therapy. Elucidating the role of STC1 will be critical for understanding the pathogenesis and identifying potential new biomarkers or therapeutic targets for OC patients.

In this study, we compared the expression levels of STC1 in para-carcinoma tissues, OC tissues and peritoneal metastasis tissues. We found that STC1 promoted metastasis in OC. Further analyses indicated that the metastasis was related to lipid metabolism. Functional studies showed that STC1 promoted lipid metabolism by up-regulating lipid-related genes such as UCP1, TOM20 and perilipin1. Mechanistically, STC1 directly bound to ITGB6 to activate the PI3K signaling pathway, and it was regulated by FOXC2. Besides, targeting STC1 and the FOXC2/ITGB6 signaling axis was related to DDP chemoresistance in OC.

## Methods

### Patients and tissue samples

After obtaining informed consent from each participant, tissue microarrays (TMAs) containing para-carcinoma tissues (*n* = 57), OC tissues (*n* = 57) and peritoneal metastasis tissues (*n* = 57) collected from patients with OC at Xinhua Hospital Affiliated to Shanghai Jiaotong University School of Medicine between 2008 and 2019 were produced. Tumor types of OC patients could be classified in epithelial tumors, sex cord stromal tumors, germ cell tumors, soft tissue tumors, unclassified type and metastatic secondary tumors [33]. The epithelial OC tumor types were selected in our study. Overall survival (OS) was measured from the date of initial surgery to the date of death from any cause or the date of the most recent follow-up. Our study was approved by the Ethics Committee of Xinhua Hospital, and each clinical investigation was conducted according to the principles outlined in the Declaration of Helsinki.

### Single-cell sequencing

Fourteen patient samples were used to conduct single-cell sequencing. The samples included peripheral blood, para-carcinoma tissues, OC tissues, peritoneal metastasis tissues, ascites and lymph nodes. Fresh para-carcinoma ovarian tissue, OC tissue and peritoneal metastasis tissue samples from patients were cut into small pieces and then digested using a MACS Tumor Dissociation Kit (Miltenyi Biotec, Bergisch Gladbach, NRW, Germany). We used marker genes CD45 and EPCAM to separate the epithelial cells from stromal cells. Cells were negative for CD45 and strongly expressed EPCAM were identified as epithelial cells [34, 35]. Cells were filtered through a seventy-millimeter cell-Strainer (BD Biosciences, San Jose, CA, USA) and centrifuged at 1200 rpm for five minutes. Then, the pelleted cells were suspended in red blood cell lysis buffer (Miltenyi Biotec, Bergisch Gladbach, NRW, Germany) to lyse red blood cells. Single cells were processed using a GemCode Gel Bead, Chip and Library Kit (10 × Genomics, Pleasanton, CA, USA) for droplet-based single-cell RNA sequencing. A total of 6000 cells were loaded for each sample. Single-cell RNA-sequencing data were quantified with Cell Ranger Single-Cell Software Suite 2.3 (10 × Genomics, Pleasanton, CA, USA).

### Cell lines and culture

The human OC cell lines Skov3-ip1 and Hey were obtained from the American Type Culture Collection (ATCC, Gaithersburg, Maryland, USA). All cells were cultured in high-glucose DMEM (Gibco, Waltham, MA, USA) supplemented with 10% fetal bovine serum (Gibco, Waltham, MA, USA), penicillin (100 U/ml) and streptomycin (100 ng/ml) and were cultured at 37 °C in a humidified atmosphere containing 5% CO_2_.

### Plasmid construction and viral infection

To generate stable cell lines, lentiviruses harboring control shRNA (negative control, NC) and specific shRNAs against STC1 (STC1-sh1/sh2), FOXC2 (FOXC2-sh1/sh2) or ITGB6 (ITGB6-sh1/sh2) (Genomeditech, Shanghai, China) were transduced into OC cells according to the manufacturers’ instructions. The sequences of NC were as follows: 5’-TTCTCCGAACGTGTCACGT-3’. The sequences of the shRNAs against STC1 were as follows: 5’-GCTGGTGATCAGTGCTTCTGC-3’ (sh1) and 5’-GCCTCAACAGTGCTCTACAGG-3’ (sh2). The sequences of the shRNAs against FOXC2 were as follows: 5’-GCGAGCAGAATTACTACCGGG-3’ (sh1) and 5’-GCTTCAGCGTGGAGAACATCA-3’ (sh2). The sequences of the shRNAs against ITGB6 were as follows: 5’-GCAACTTTAGACTGGGCTTCG-3’ (sh1) and 5’-GCCAACCCTTGCAGTAGTATT-3’ (sh2). Briefly, the OC cell lines Skov3-ip1 and Hey were infected by incubating cells with medium containing virus and 1 ng/ml polybrene (Sigma, ST LOUIS, MO, USA) for 24 h. Then, stably transduced cells were screened using puromycin (Sigma) at a final concentration of 2 mg/ml for 3 days. Plasmid DNA was transfected into cells with Lipofectamine 2000 (Invitrogen, Waltham, MA, USA).

### RNA sequencing data analysis

RNA sequencing assays were performed as previously described [36]. Briefly, total RNA (1 ug) was isolated from OC tissues and peritoneal metastasis tissues and incubated with VAHTS mRNA Capture Beads (Vazyme, Nanjing, China) to enrich polyA + RNA before construction of RNA libraries. Computational analysis of RNA sequencing data was performed with the Ensemble Human Genome Assembly (Genome Reference Consortium GRCh38) as the reference genome. The expression levels for each gene transcript were estimated as the number of reads per kilobase of exon model per million mapped reads (RPKM). Gene set enrichment analysis (GSEA) were used for gene functional annotation. Hallmark collections were acquired from the Molecular Signatures Database (MSigDB), and were analyzed by GSEA with GSEA software (version: 4.0.3). A total of 5000 permutations were performed, based on the gene set, to determine p-values. Pathways with p < 0.01 and false discovery rate (FDR) < 0.25 were considered significant. “Signal to noise” was used as a metric for ranking the genes.

### Immunohistochemical (IHC) assay

The TMAs were produced by Xinhua Hospital affiliated to Shanghai Jiaotong University School of Medicine. IHC staining was performed on 7-mm-thick TMA sections. The sections were deparaffinized and rehydrated in a descending ethanol series. After antigen retrieval, the sections were incubated with 3% hydrogen peroxide for 20 min. Tissue slides were then incubated for 12 h at 4 °C with primary antibodies specific for the following proteins: STC1 (ab229477, Abcam, 1:100 dilution), FOXC2 (AF6989-SP, R&D, 1:100 dilution) and ITGB6 (10,176–2-AP, Proteintech, 1:100 dilution). The next day, after incubation with a horseradish peroxidase-labeled secondary antibody for 30 min at room temperature, the sections were rinsed three times with phosphate buffer saline (PBS) for 5 min each. Approximately 5 ml of diaminobenzidine (DAB) was added for 3–15 min to induce color development. The sections were then rinsed thoroughly with tap and were then counterstained, dehydrated, cleared with xylene, covered with neutral resin, and observed under a microscope. The antibodies used are listed in Supplementary Table S1 (The primary antibodies).

### Untargeted lipidomics and proteomics

Lipids from cells were extracted in a methanol: MTBE: chloroform (MMC) mixture (40/30/30, v/v/v), containing the antioxidant BHT (10 μg/100 ml). Each sample was then vortexed and placed in a shaker at room temperature for 30 min (950 rpm). Samples were then centrifuged to pellet proteins (10 min, 8000 rpm), and the supernatant saved for analysis in fresh Eppendorf tubes. A volume of two μl of each sample was injected into the LC–MS instrument. All Dionex Ultimate 3000 series modules (Thermo Fisher Scientific, Waltham, MA, USA) and a Thermo Q-Exactive mass spectrometer (Thermo Fisher Scientific, Waltham, MA, USA) were used. Liquid chromatographic separation was performed at 45 °C using a Kinetex F5 reversed-phase column (Phenomenex Inc.), at a flow rate of 0.65 ml/min. The mobile phases consisted of 5 mM ammonium formate and 0.1% formic acid in water and 5 mM ammonium formate and 0.1% formic acid in isopropanol. Mass spectrometry analysis was performed first with positive/negative ion switching method in full MS scan mode. Preliminary untargeted data analysis with Lipostar software (Molecular Discovery Ltd., UK) allowed automatic generation of inclusion lists for masses of interest (i.e., potential lipids). Finally, MS/MS spectra were uploaded in the Lipostar session to improve lipid identification, and the same software was used for multivariate statistical data analysis.

### Transmission electron microscopy (TEM)

For TEM observation, cells subjected to different treatments were fixed with 5% glutaraldehyde (in PBS buffer) at 4 ℃ for 12 h. After fixation, cells were washed with PBS, and fixed again for 2 h at 4 °C in aqueous 1% (w/v) osmium tetroxide and then embedded in Epon. Observation and imaging were carried out with an electron microscope (JEM-2000EX TEM, JEOL, Tokyo, Japan).

### Oil Red O (ORO) Staining

After 8 days of differentiation, cells in different groups were stained with ORO (#O0625, Sigma, USA). Staining was performed according to the manufacturer's instructions. To quantify the staining of fat droplets, 100% isopropanol was used to dissolve the lipid droplets, and the absorbance was measured at 510 nm.

### Immunofluorescence (IF) assay

Cells or frozen sections of OC tissues were fixed with 4% paraformaldehyde for fifteen minutes, permeabilized with 0.3% Triton X-100 for 15 min, and blocked with 5% goat serum (Life Technologies, USA) for 1 h at room temperature. Then, sections were incubated with primary antibodies for 12 h at 4 °C. Secondary antibodies were added, and staining with DAPI (Life Technologies, USA) was then performed. Stained sections were imaged using a Leica SP5 confocal fluorescence microscope. Primary antibodies specific for the following proteins were used in the IF experiments: STC1 (sc-293435, Santa Cruz Biotechnology, 1:100 dilution, Dallas, Texas, USA), FOXC2 (23,066–1-AP, Proteintech, 1:100 dilution, Wuhan, Hubei, China), ITGB6 (19,695–1-AP, Proteintech, 1:100 dilution, Wuhan, Hubei, China), UCP1 (AF8292, Beyotime, 1:100 dilution, Shanghai, China), TOM20 (11,802–1-AP, Proteintech, 1:100 dilution, Wuhan, Hubei, China), perilipin1 (ab172907, Abcam, 1:100 dilution, Cambridge, UK). All antibodies are listed in Supplementary Table S1 (The primary antibodies). The secondary antibodies were FITC- or Texas red-conjugated donkey F(ab)2 fragments specific for mouse IgG or rabbit IgG (Jackson ImmunoResearch Laboratories).

### Western blotting (WB) assay

WB analysis was performed as previously described [37]. Briefly, cells were harvested and lysed in RIPA buffer in the presence of protease inhibitor cocktail (Pierce, WA, USA) and a protein phosphatase inhibitor (Beyotime, Shanghai, China). One hundred micrograms of protein was separated by 10% SDS-PAGE and transferred onto PVDF membranes (Tanon, Shanghai, China). Membranes were blocked with 5% bovine serum albumin (BSA) for 2 h and were then incubated for 12 h with primary antibodies at 4 °C. Membranes were probed with antibodies specific for STC1 (20,621–1-AP, Proteintech, 1:1000 dilution, Wuhan, Hubei, China), FOXC2 (23,066–1-AP, Proteintech, 1:1000 dilution, Wuhan, Hubei, China), p-FAK (8556S, CST, 1:1000 dilution, Boston, MA, US), p-AKT (86,444–1-Ig, Proteintech, 1:1000 dilution, Wuhan, Hubei, China), p-PI3K (17366 s, CST, 1:1000 dilution, Boston, USA), PI3K (20,584–1-AP, Proteintech, 1:1000 dilution, Wuhan, Hubei, China), FAK (12,636–1-AP, Proteintech, 1:1000 dilution, Wuhan, Hubei, Chian), AKT (10,176–2-AP, Proteintech, 1:1000 dilution, Wuhan, Hubei, China), ITGAV (27,096–1-AP, Proteintech, 1:1000 dilution, Wuhan, Hubei, China), ITGB6 (19,695–1-AP, Proteintech, 1:1000 dilution, Wuhan, Hubei, China), ITGB7(A5873, Abclonal, 1:1000 dilution, Wuhan, Hubei, China). GAPDH (10,494–1-AP, Proteintech, 1:5000 dilution, Wuhan, Hubei, China) was used as the internal control. Goat anti-mouse or goat anti-rabbit horseradish- peroxidase-conjugated IgG was used as the secondary antibody (Beyotime, Shanghai, China) at a 1:1000 dilution. Membranes were incubated with the secondary antibody for 1 h at room temperature, and immunoreactive bands were visualized using an enhanced chemiluminescence detection system (Amersham Bioscience, Piscataway, NJ, USA) according to the manufacturer’s instructions. Three independent experiments were performed for final analyses.

### Cell proliferation assay

Cell proliferation assay was tested with a CCK-8 kit (Beyotime, Shanghai, China, Cat# C0039). The different groups of cells were seeded in 96-well plates at a density of 2000 cells per well. After culturing for 0, 24, 48, 72, 96, and 120 h, 200 μl of medium containing CCK-8 reagent was prepared, added to the wells, and incubated for two hours. The absorbance values were measured at a wavelength of 450 nm, with a microplate reader (BioTeke, Beijing, China).

### Cell migration and invasion assay

Starved cells (5 × 10^5^ for Skov3-ip1 cells, 3 × 10^5^ for Hey cells) were plated in the upper compartment of Transwell chambers in 250 μl of serum-free medium. Complete medium containing 10% FBS was added to the bottom compartment. Plates were incubated in 5% CO_2_ at 37 °C for 12 h. Then, the cells remaining in the upper compartment were removed with a cotton swab, and the migrated cells were fixed with paraformaldehyde and stained with crystal violet. The invasion experiment required the membrane in the upper compartment to be coated with Matrigel before cell seeding. Images acquired, and some of the stained cells were counted under a microscope.

### Colony formation assay

Cells were seeded in six-well plates at a density of 500 cells per well. Cells were incubated at 37 °C in 5% CO_2_ and allowed to grow for at least 10 days, the medium was then discarded. Colonies were counted after being fixed with paraformaldehyde (4%) for 15 min and were stained with crystal violet (Beyotime, Shanghai, China) for 30 min.

### Wound healing assay

A wound healing assay was used to measure the cell migration ability of Skov3-ip1 and Hey cells under specific conditions. The different groups of cells were plated in six-well plates and allowed to grow to confluence. A wound was made by scratching the cell monolayers with a 200 μl pipette tip. The indicated cells were deprived of serum for 48 h. Images of migration were acquired at 0 h and 12 h after scraping.

### Oxygen consumption rate (OCR) and extracellular acidification rate (ECAR) measurement

The ECAR and OCR were measured using a Seahorse XF Glycolysis Stress Test Kit (Agilent Technologies, Palo Alto, CA, USA) and a Seahorse XF Cell Mito Stress Test Kit (Agilent Technologies, Palo Alto, CA, USA) and were analyzed using a Seahorse XF24 analyzer (Agilent Technologies, Palo Alto, CA, USA). Briefly, glucose uptake of STC1-sh1/sh2 and NC cells of the two cell lines was detected. These cells were harvested 48 h after seeding, and used to measure the ECAR and OCR. After baseline measurements, for OCR measurement, 1 μmol/L oligomycin, 1 μmol/L FCCP (p‐trifluoromethoxy carbonyl cyanide phenylhydrazone, a reversible inhibitor of oxidative phosphorylation), and 0.5 μmol/L Rote/AA (rotenone plus the mitochondrial complex III inhibitor antimycin A were automatically injected successively. For ECAR measurement, the Seahorse automatically filled each well with 10 mmol/L glucose, 1 μmol/L oligomycin (an oxidative phosphorylation inhibitor), and 50 mmol/L 2‐DG (2‐deoxy‐D‐glucose, a glycolysis inhibitor) successively. Data were analyzed by using Seahorse XF24 Wave software (Agilent Technologies, Palo Alto, CA, USA). The OCR and ECAR values were calculated after normalization to the cell number and were plotted as the mean SD values.

### Immunoprecipitation (IP)

IP experiments were performed as previously described [38, 39]. Briefly, cells were extracted with RIPA lysis buffer containing phosphatase and protease inhibitors. Cell lysates were incubated with 1 μg indicated antibodies and protein A-Sepharose (GE Healthcare). The cell lysates, antibodies and sepharose mix were incubated at 4 °C overnight. Then wash the immunocomplexes four times with lysis buffer and analyzed by WB assays. Antibody used was as follow: STC1 (ab229477, Abcam).

### Co-immunoprecipitation (co-IP)

Cell extracts were incubated with 0.5 μg individual antibody and 20 μl protein G beads (GE Healthcare, Pittsburgh, USA). After overnight incubation, beads were washed four times with lysis buffer, separated by SDS-PAGE, and analyzed by immunoblotting.

### Chromatin Immunoprecipitation (ChIP) assay

ChIP assays were performed using a Pierce Agarose ChIP Kit (Thermo, #27,177) following the manufacturer’s guidance. Briefly, Skov3-ip1 cells were cross-linked with 1% formaldehyde for 10 min at 37 °C. The cross-linking reaction was quenched with glycine, and cells were lysed in SDS buffer containing protease inhibitor cocktail. Cell lysates were sonicated to shear chromatin into fragments with a length of 200–1000 base pairs and were then subjected to immunoprecipitation with 4 μl of IgG (Cell Signaling Technology), 10 μl of anti-FOXC2 (ab5060, Abcam), or 3 μl of anti-Polymerase II (Imgenex) antibodies. After washing with a series of low- and high-salt washing buffers, immunoprecipitated DNA fragments were decrosslinked at 77 °C under high-salt conditions and purified using a QIAquick PCR purification kit (Qiagen). The amount of DNA was further assessed by qRT-PCR using FOXC2-specific ChIP primers and SYBR Select Master Mix (Applied Biosystems, Grand Island, NY, USA). The primer sequences were as follows: F, 5’- GGCTTGAACACAGATTATTA -3’ and R, 5’- TGAGATATGACAGCTCAGAG -3’.

### qRT-PCR

Total RNA was extracted from cells with TRIzol reagent (Invitrogen, Carlsbad, CA, USA), and it was reverse transcribed using a miScript Reverse Transcription Kit (Qiagen, Dusseldorf, NRW, Germany). The primers used for mRNA quantification are listed in Supplementary Table S2 (The primer sequences of qRT-PCR assay). Quantification was performed with a QuantiTect Probe RT-PCR kit (Qiagen, Dusseldorf, NRW, Germany). The comparative threshold cycle method was used to determine relative gene expression levels.

### Förster resonance energy transfer and fluorescence lifetime imaging (FRET-FLIM)

For the FRET-FLIM experiments, donor proteins (fused to GFP) were expressed by the vector pCMV3-C-GFP Spark, and acceptor proteins (fused to RFP) were expressed by the vector pCMV3-C-RFP Spark. FRET-FLIM experiments were performed with a Leica TCS SMD FLCS confocal microscope. Skov3-ip1 or Hey cells transiently coexpressing the donor and acceptor proteins, as indicated in the figures, were visualized thirty-six hours after transfection. Accumulation of GFP-tagged and RFP-tagged proteins was estimated before measuring the fluorescence lifetime. A tunable WLL set at 489 nm with a pulsed frequency of 40 MHz was used for excitation, and emission was detected using a SMD GFP/RFP filter cube. The fluorescence lifetime shown in the figures corresponding to the average fluorescence lifetime of the donor (τ) was determined and analyzed with PicoQuant SymphoTime software. The lifetime is generally defined as the amplitude-weighted mean value using data from the single (GFP-fused donor protein only) or biexponential (the GFP-fused donor protein interacting with the RFP-fused acceptor protein) fit. Mean lifetimes are presented as the means ± SDs based on more than ten cells from at least three independent experiments. The FRET efficiency was evaluated according to the formula E = 1-τDA/τD. τDA is the average lifetime of the donor in the presence of the acceptor, and τD is the average lifetime of the donor in the absence of the acceptor.

### Animal experiments

Female athymic nude mice (4–6 weeks old) weighing 14–16 g were purchased from Xinhua Hospital Affiliated to Shanghai JiaoTong University School of Medicine and bred under specific pathogen-free conditions. Prior to the study, the protocols for the treatment of animals were approved by the Medical Animal Care Committee. A total of 5 × 10^5^ Skov3-ip1 or Hey human OC cells transfected with luciferase reporter vectors (Skov3-ip1-Luc/Hey-Luc) in 10 μl of serum-free DMEM were injected into the left ovarian parenchyma of the nude mice. Each group contained five mice. Mice were monitored every five days for tumor growth, and tumor size was measured using a caliper. After four weeks, mice were euthanized, and the left ovary and enterocele in the regions showing clear luciferase signals were excised. The total flux, weight, and the distribution of the tumor were recorded. Tumor tissues were fixed in 4% neutral buffered formalin for frozen slide preparation.

### H&E staining

Consecutive tissue sections (thickness, 5 μm) of paraffin-embedded tumor specimens were prepared. Tissue sections were stained with hematoxylin for 5 min and rinsed with running water for 5 min. Next, they were soaked in hydrochloric acid solutions for 5 s, rinsed with running water for another 10 min, and then immersed in ammonia for 5 s. Tissue sections were then rinsed with running water for 10 min, stained with eosin solution for 30 s, and briefly immersed in distilled water. Last, the sections were rapidly dehydrated in graded ethanol (80%, 95%, and 100%), and mounted with neutral gum.

### Drug treatment and apoptosis detection

Cells were treated with 50 µg/ml DDP (MCE, Newark, NJ, USA). Apoptosis induced by DDP was assessed via flow cytometry after cells were stained with propidium iodide (PI) and Annexin V (Beyotime, Shanghai, China). Briefly, 1 × 10^6^ cells seeded in 60 mm dishes for 24 h were treated with DDP for 12 h and were then trypsinized and washed twice in ice-cold PBS. A total of 1 × 10^5^ cells were resuspended in 100 µl of binding buffer to which 5 µl of 2 mg/ml Annexin V and 5 µl of 50 µg/ml PI were added. After 15 min of incubation in the dark, cells were evaluated by flow cytometry (BD Biosciences, Franklin Lakes, NJ, USA) in different channels, and Cell Quest software version 3.3 (BD Biosciences) was used for analysis. All experiments were repeated three times.

### Statistical analysis

Differences between two groups were analyzed using Student’s t test and one-way analysis of variance for normally distributed data and using the Mann–Whitney U test for nonnormally distributed data. A *P*-values < 0.05 was set as the significance threshold. Before the survival analysis, the mean expression level was used to classify patient samples into two groups. Kaplan–Meier survival analysis was performed to assess the associations of gene expression levels with clinical outcomes, and *P*-values were calculated with the log-rank test.

## Results

### STC1 is closely associated with OC metastasis and the metastasis is related to lipid metabolism

**Fig. 1:**
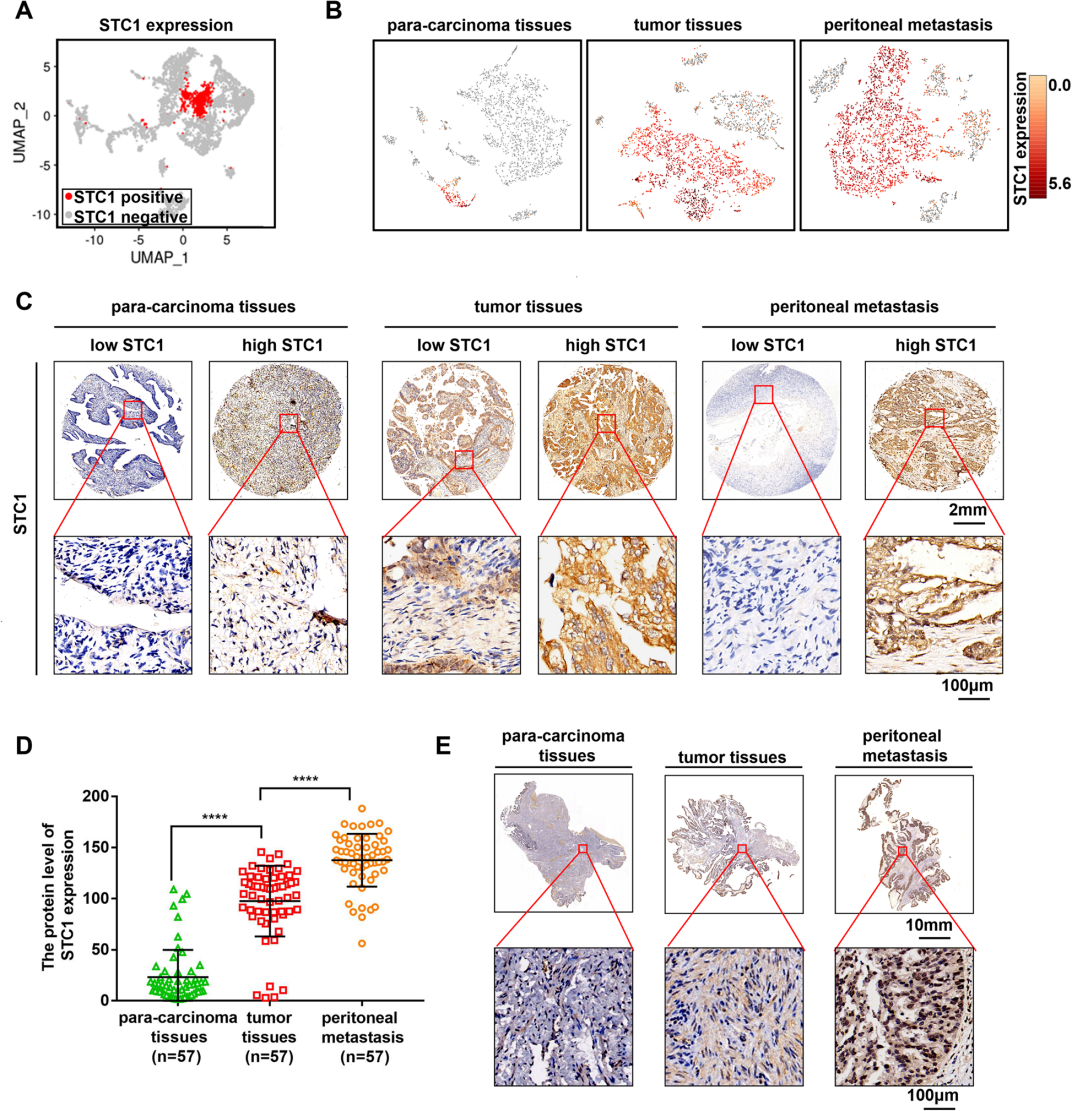
STC1 is closely associated with OC metastasis and the metastasis is related to lipid metabolism. **A** Single-cell sequencing results of STC1 in patient tissues (red region: high expression of STC1). **B** Single-cell sequencing results of STC1 expression (red region) in para-carcinoma tissues, tumor tissues and peritoneal metastasis tissues of OC patients. **C** Representative images of IHC staining from TMAs of STC1 in para-carcinoma tissues, tumor tissues and peritoneal metastasis tissues of OC patients. **D** The protein level of STC1 expression in para-carcinoma tissues, tumor tissues and peritoneal metastasis tissues of OC patients. **E** Representative pictures of IHC assay of STC1 in para-carcinoma tissues, tumor tissues and peritoneal metastasis tissues. (Data are shown as the mean ± SD values. Significance was calculated using Student’s t test. *, *P* < 0.05; **, *P* < 0.01; ***, *P* < 0.001; ****, *P* < 0.0001

We conducted single-cell sequencing of OC tissues and found that STC1 was highly expressed in some cells (red region) (Fig. 1A). Then, we found that the cells in red region mainly concluded epithelial cells, which indicated that STC1 was highly expressed in epithelial cells in OC tissues (Supplementary Fig. 1A (Enrichment analysis of STC1 in different types of cell subgroups through single-cell sequencing)). Furthermore, we found that the expression of STC1 was up-regulated in OC tissues compared with para-carcinoma tissues and was further increased in peritoneal metastasis tissues (red region) (Fig. 1B). After that, we conducted an IHC assay of three types of tissues from TMAs and determined that STC1 expression was higher in tumor and peritoneal metastasis tissues than in para-carcinoma tissues, which verified the results of single-cell sequencing (Fig. 1C-D). Similarly, we conducted another IHC assay, which proved that STC1 expression was negative or minimal in para-carcinoma tissues, while it was specific and positive in tumor and peritoneal metastasis tissues (representative pictures shown in Fig. 1E). Since the most common subtype of OC is epithelial OC (EOC) [40], we indicated that STC1 was highly expressed in OC tissues, which implied that STC1 was highly expressed in OC tumor cells, and the high expression of STC1 was related to metastasis in OC tissues. In addition, we performed RNA sequencing of OC tissues and peritoneal metastasis tissues, and then we conducted Gene Ontology (GO) analysis and GSEA (Supplementary Fig. 1B-C (GO analysis of tumor tissues and peritoneal metastasis tissues of OC patients. GSEA of peritoneal metastasis tissues and tumor tissues of OC patients)). The results showed that some genes involved in fatty acid metabolism were enriched. Hence, we found that STC1 was highly associated with metastasis in OC and such metastasis was related to lipid metabolism. Subsequently, we obtained the medical history of 114 patients with EOC and analyzed their clinicopathological characteristics as well as their STC1 expression levels (Supplementary Table S3 (Association of STC1 expression with clinicopathological characteristics in 114 patients of EOC)). A high level of STC1 was positively correlated with advanced tumor stage (stage III/IV) and high tumor grade (grades 2–3), while a low level of STC1 was positively correlated with early tumor stage (stage I/II) and low tumor grade (grades 1). These results suggest that STC1 is a potential prognostic biomarker in OC patients and that its expression is highly predictive of the clinical outcome. Overall, we indicated that STC1 was closely associated with metastasis in OC tissues and the metastasis was relate to lipid metabolism.

### STC1 promotes metastasis and lipid metabolism in vitro

**Fig. 2:**
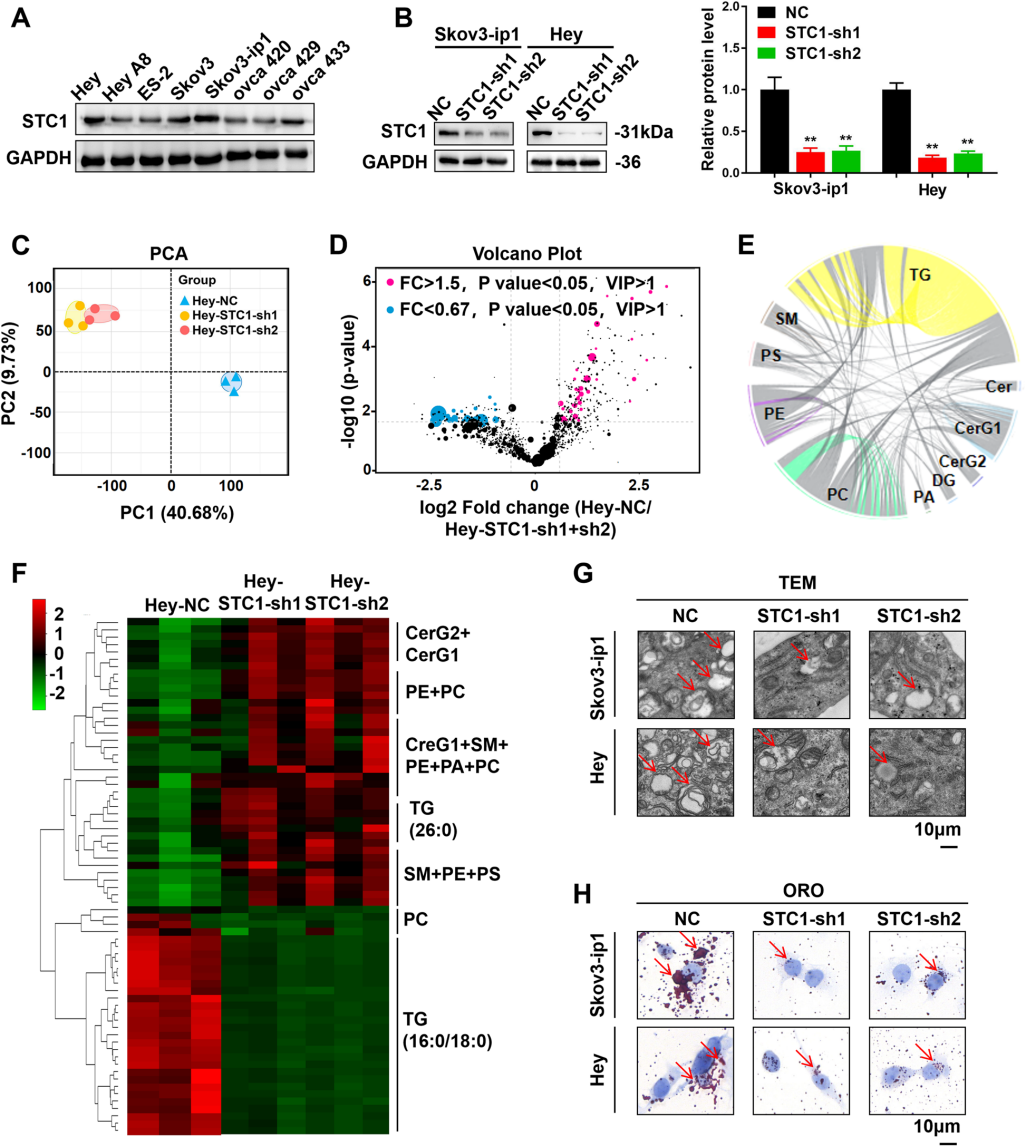
STC1 promotes metastasis and lipid metabolism in vitro. **A** WB results of the STC1 expression levels in different OC cell lines. **B** STC1-knockdown cell lines were established with Skov3-ip1 and Hey cells, and the transfection efficiency was confirmed by WB assays.** C** PCA of data from untargeted relative quantitative lipidomic analysis. **D** Volcano plot of individual lipid species significantly up- or down- regulated in the NC group versus the STC1-sh1 and STC1-sh2 groups in Hey cell lines (lipids with a *P* value < 0.05 and a fold change (FC) > 1.5 or < 0.67 were indicated in blue or red, respectively). **E** Correlation analysis of the ten lipid classes. **F** A heat map of significant differences in the variables among the NC, STC1-sh1 and STC1-sh2 groups of Hey cells. The variables were ranked by ANOVA. **G** TEM images of OC cells in Skov3-ip1 and Hey cell lines (red arrow: mitochondria). **H** ORO staining of OC cells in Skov3-ip1 and Hey cell lines (red arrow: TGs). (Data are shown as the mean ± SD values. Significance was calculated using Student’s t test. *, *P* < 0.05; **, *P* < 0.01; ***, *P* < 0.001; ****, *P* < 0.0001.)

As described above, STC1 expression was significantly up-regulated in OC tissues, especially in peritoneal metastasis tissues. Therefore, we further investigated the role of STC1 in metastasis. First, we found that Skov3-ip1 and Hey cells had the highest expression of STC1 among several OC cell lines (Fig. 2A). Then, two shRNAs were designed, and Skov3-ip1 and Hey cell lines with stable STC1 knockdown (STC1-sh1 and STC1-sh2) were constructed via lentiviral transduction (Fig. 2B). The results of cell proliferation experiments showed that the cell proliferation ability was down-regulated in the STC1-sh1 and STC1-sh2 groups compared with the NC groups of the two cell lines, which indicated that knockdown of STC1 could decrease the cell proliferation ability of OC cells (Supplementary Fig. 2A (Cell proliferation assays in Skov3-ip1 and Hey cell lines transfected with the NC groups, STC1-sh1 and STC1-sh2 groups)). Next, the results of colony formation assays showed that the colony formation rate was lower in the STC1-sh1 and STC1-sh2 groups than in the NC groups of the two cell lines, which demonstrated that knockdown of STC1 could decrease the colony formation ability of OC cells (Supplementary Fig. 2B-C (Colony formation assays in Skov3-ip1 and Hey cell lines and statistical analysis)). After that, the results of cell migration and invasion assays showed that cell migration and invasion were decreased in the STC1-sh1 and STC1-sh2 groups compared with the NC groups of the two cell lines, which indicated that knockdown of STC1 could decrease the migration and invasion of OC cells (Supplementary Fig. 2D-F (Cell migration and invasion assays in Skov3-ip1 and Hey cell lines and statistical analysis)). Additionally, the results of wound healing experiments showed that the migration ability was decreased in the STC1-sh1 and STC1-sh2 groups compared with the NC groups of the two cell lines, which further demonstrated that knockdown of STC1 could decrease the cell migration ability in OC cells (Supplementary Fig. 2G (Wound healing assays in Skov3-ip1 and Hey cell lines and statistical analysis)). These results showed that knockdown of STC1 weakened the proliferation and metastasis ability in vitro.

Then, we explored the relationship between STC1 expression and lipid metabolism. We conducted untargeted relative quantitative lipidomic assays using STC1-sh1 and STC1-sh2 Hey cells and their corresponding controls. Systematic lipidomic changes occurring in different groups were then assessed by principal component analysis (PCA), a widely used multivariate method (Fig. 2C). A total of 1412 lipid species from 30 lipid classes were identified, and 70 of them exhibited significant differences (Supplementary Fig. 3A (A total of 1412 lipid species from 30 lipid class were identified)). To summarize the individual lipid species significantly up- or down- regulated in control cells compared to STC1-sh1 and STC1-sh2 cells, volcano plots showed these lipids were generated and were presented in Fig. 2D. Lipids with a P value < 0.05 and a fold change (FC) > 1.5 or < 0.67 were indicated in blue or red, respectively. Relative quantifications were next performed for all the identified lipids, and 70 significantly changed lipids from 10 lipid classes were presented in Supplementary Fig. 3B (70 significantly changed lipids from 10 lipid classes were presented). The results of the correlation analysis of the 10 lipid classes were presented in Fig. 2E. Metabolomics analyses were next performed. Significant differences in the variables among the control group, STC1-sh1 group and STC1-sh2 group were depicted as a heat map in Fig. 2F, and were ranked by ANOVA. Each row represented an individual lipid, and each column represented an individual sample. Interestingly, correlation values among the 70 lipids revealed 2 distinct clusters. Obviously, in the STC1-sh1 and STC1-sh2 groups, many triglycerides (TGs) (such as 16:0 and 18:0) were down-regulated, as were some phosphatidyl cholines (PCs) (such as 36:0) and ceramides (Cers) (such as d18:1). In addition, a small number of TGs (such as 26:0) were up-regulated. Collectively, our lipidomic data revealed a strikingly consistent separation in OC cells. In addition, we visualized the microstructures of OC cells using TEM. We found that there were more mitochondria (red arrow) in the NC group than in the STC1-sh1 and STC1-sh2 groups (Fig. 2G). Moreover, a large number of pseudopodia and protuberances could be seen around the cell membrane in the NC groups, and the swelling was relatively severe. These results indicated that in OC cells, lipid accumulation was enhanced. We also performed ORO staining in OC cells, which showed that large amounts of TGs (red arrow) accumulated in the cytoplasm in the cells of the NC groups (Fig. 2H). Overall, these findings suggested that STC1 promoted metastasis and lipid metabolism in vitro and that TGs may be involved in this process.

### STC1 promotes lipid metabolism by regulating lipid-related genes in vitro

**Fig. 3:**
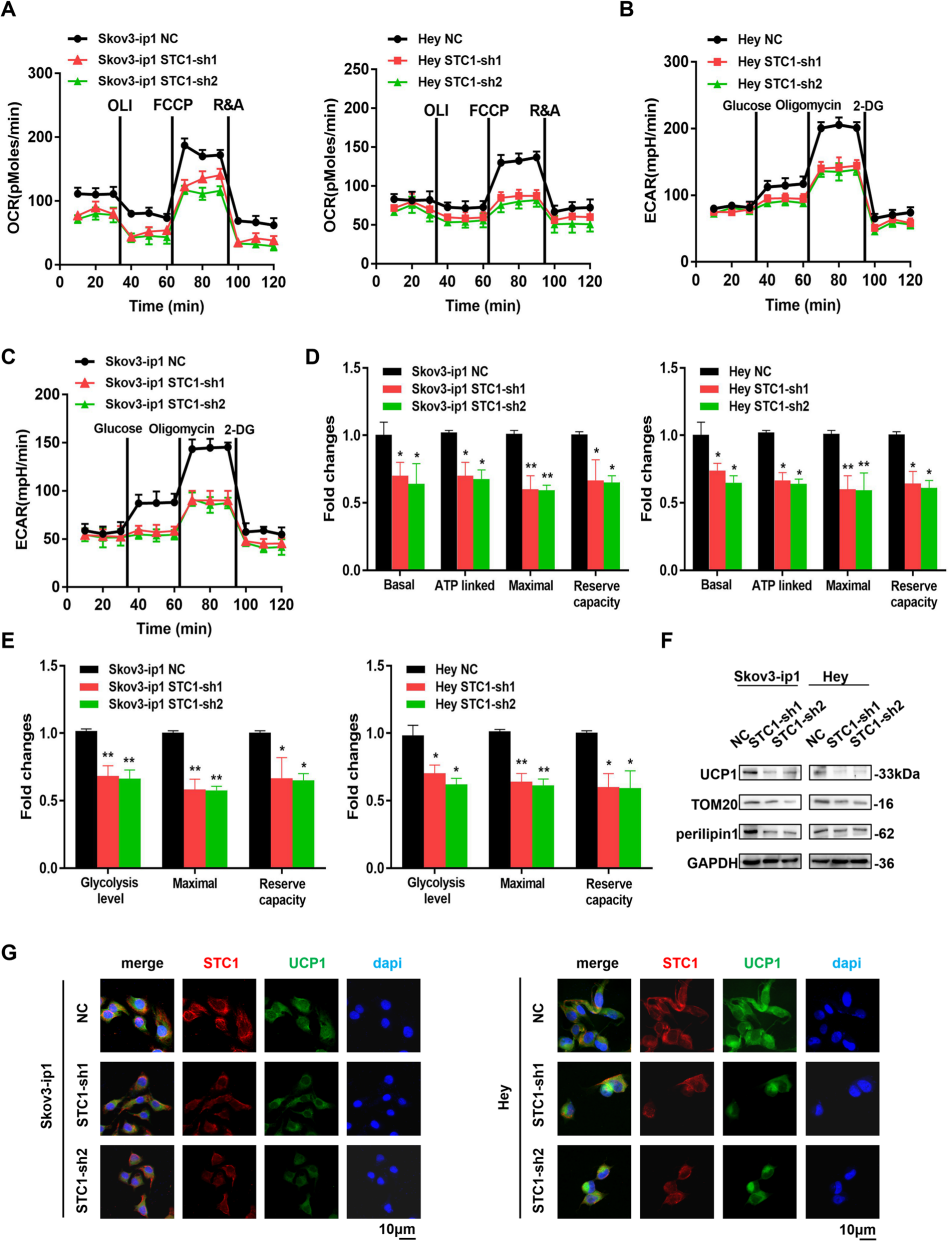
STC1 promotes lipid metabolism by regulating lipid-related genes in vitro. **A-E** The OCR, ECAR results and related statistical analysis in Skov3-ip1 and Hey cell lines. **F** Representative WB results of UCP1, TOM20 and perilipin1 in Skov3-ip1 and Hey cell lines. **G** IF analysis showed the relationship between STC1 and UCP1 expression in Skov3-ip1 and Hey cell lines. (Data are shown as the mean ± SD values. Significance was calculated using Student’s t test. *, *P* < 0.05; **, *P* < 0.01; ***, *P* < 0.001; ****, *P* < 0.0001.)

Since lipids are necessary elements in OC metastasis and provide cancer cells with energy, we demonstrated that STC1 promoted lipid metabolism by regulating lipid-related genes. To investigate the mechanism of action of STC1 in mitochondrial biogenesis and modulation of lipid droplet size, we evaluated overall mitochondrial function using a Seahorse XF24 Extracellular Flux Analyzer to measure the OCR and ECAR in Skov3-ip1 and Hey cell lines (Fig. 3A-C). The basal oxygen consumption, ATP-linked OCR, maximal respiration and reserved capacity were decreased in STC1-sh1 and STC1-sh2 cells compared with those in control cells (Fig. 3D). In addition, the glycolysis level and reserved capacity were also decreased in STC1-sh1 and STC1-sh2 cells compared with those in control cells (Fig. 3E). In parallel, the expression levels of a mitochondrial proteins (UCP1), outer mitochondrial membrane-20 (TOM20) and a lipid droplet-binding protein (perilipin1) were decreased in STC1-sh1 and STC1-sh2 cells compared with those in control cells, as shown by WB analysis in the two cell lines (Fig. 3F). IF analyses confirmed the down-regulation of UCP1, TOM20 and perilipin1 in STC1-sh1 and STC1-sh2 cells compared with those in control cells (Fig. 3G and Supplementary Fig. 3C-D (IF assays detected the relationship between STC1 and TOM20 in Skov3-ip1 and Hey cell lines. IF assays detected the relationship between STC1 and perilipin1 in Skov3-ip1 and Hey cell lines)). In summary, we demonstrated that STC1 promoted lipid metabolism by up-regulating lipid-related genes such as UCP1, TOM20 and perilipin1 in vitro.

### STC1 directly binds to ITGB6 to activate the PI3K signaling pathway, participating in lipid metabolism in vitro

**Fig. 4:**
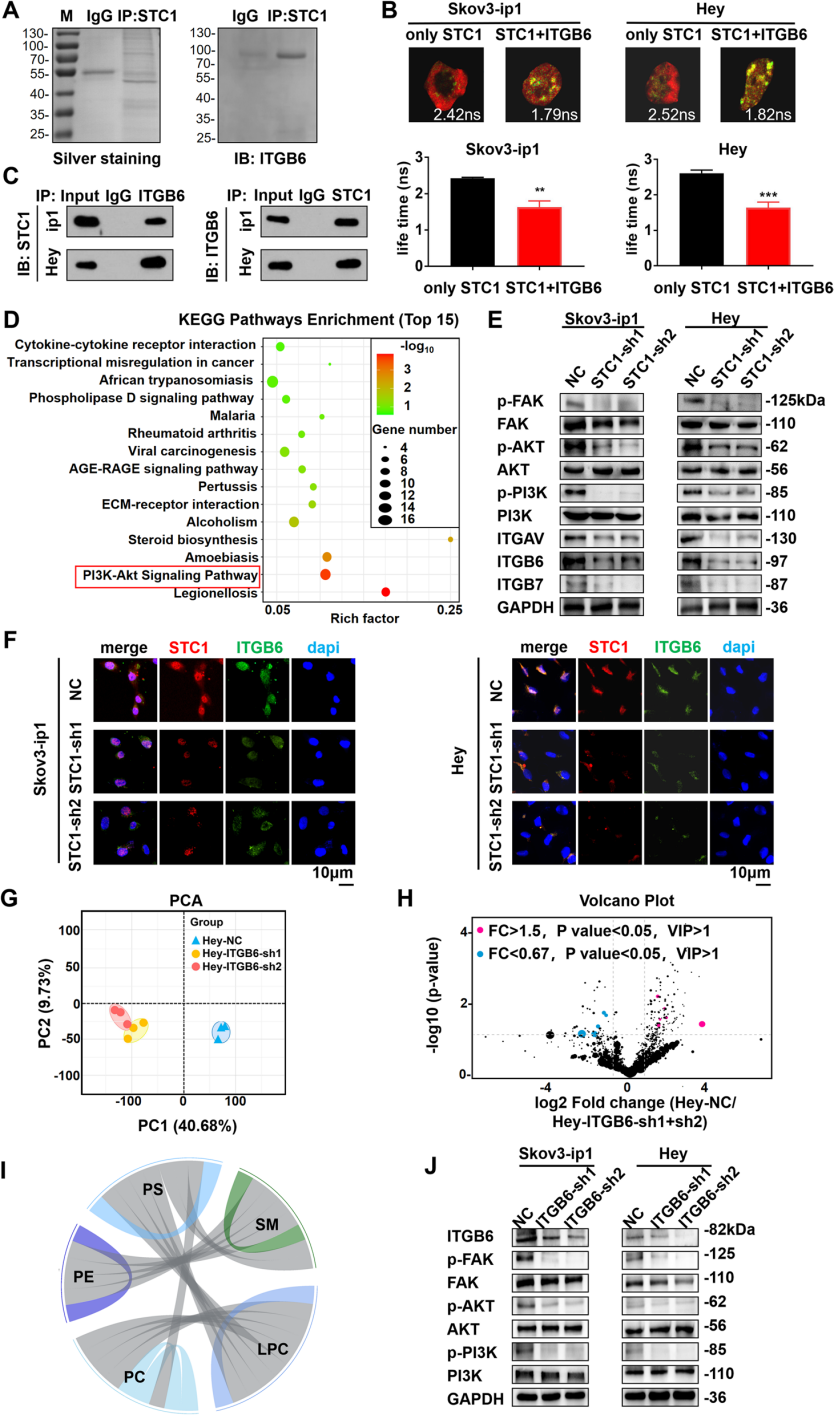
STC1 directly binds to ITGB6 to activate the PI3K signaling pathway, participating in lipid metabolism in vitro. **A** Cell extracts from Skov3-ip1 cells were immunopurified on STC1 affinity columns and eluted with the STC1 peptide. The eluates were resolved by SDS-PAGE and silver staining, and the results were confirmed by WB assays. **B** The interaction between STC1 and ITGB6 was confirmed by FRET-FLIM upon transient co-expression. **C** Co-IP analysis demonstrated an interaction between the STC1 and ITGB6 proteins in Skov3-ip1 and Hey cell lines. **D** Top 15 pathways identified by KEGG pathway enrichment analysis. **E** Representative WB results of proteins in the PI3K signaling pathway and integrin family in Skov3-ip1 and Hey cell lines. **F** IF analysis identified the co-localization of STC1 and ITGB6 in Skov3-ip1 and Hey cell lines. **G** PCA of data from untargeted relative quantitative lipidomic analysis. **H** Volcano plot of individual lipid species significantly up- or down- regulated in the NC group versus the ITGB6-sh1 and ITGB6-sh2 groups in Hey cell lines (lipids with a *P* value < 0.05 and a fold change (FC) > 1.5 or < 0.67 were indicated in blue or red, respectively). **I** Correlation analysis of the 5 lipid classes. **J** Representative WB results of ITGB6 and proteins in the PI3K signaling pathway in Skov3-ip1 and Hey cell lines. (Data are shown as the mean ± SD values. Significance was calculated using Student’s t test. *, *P* < 0.05; **, *P* < 0.01; ***, *P* < 0.001; ****, *P* < 0.0001.)

In order to investigate the potential proteins associated with STC1, we conducted IP assay and mass spectrometry analyses. We found that STC1 was related to ITGB6, an integral cell surface protein that participates in cell adhesion as well as cell surface-mediated signaling (Fig. 4A and Supplementary Fig. 4A (Mass spectrometry analysis of the SDS-PAGE above. The detected peptides spectra of ITGB6 were listed)). Moreover, the results of FRET-FLIM further demonstrated the direct interaction between STC1 and ITGB6 (Fig. 4B). Then, we conducted co-IP assays, which showed that STC1 directly bound to ITGB6 (Fig. 4C). Additionally, Kyoto Encyclopedia of Genes and Genomes (KEGG) pathway enrichment analysis showed that the PI3K-Akt signaling pathway was related to STC1 (Fig. 4D). Next, we conducted WB analysis and found that stable knockdown of STC1 decreased the expression levels of phosphorylated PI3K, FAK and AKT, and it also decreased the expression levels of the members of the integrin family (ITGAV, ITGB6 and ITGB7) (Fig. 4E). The IF assay demonstrated the co-localization of STC1 and ITGB6 (Fig. 4F). To further address the relationship between STC1 and ITGB6, we conducted rescue experiments by transfecting the ITGB6 OE plasmid into two cell lines (Supplementary Fig. 4B (The knockdown of STC1 expression level in the STC1-sh1 groups were rescued by ITGB6 OE plasmid in Skov3-ip1 and Hey cell lines)). We found that after transfection of the ITGB6 OE plasmid into the STC1-sh1 group, the protein expression of STC1 was up-regulated accordingly in the two OC cell lines, which demonstrated that ITGB6 interacted with STC1. Next, the results of cell proliferation assays showed that after transfection of the ITGB6 OE plasmid into the STC1-sh1 groups, the cell proliferation ability was up-regulated compared with that of the STC1-sh1 groups and was not significantly different compared with that of the NC groups (Supplementary Fig. 4C (Cellproliferation assays in Skov3-ip1 and Hey cell lines in the NC groups, STC1-sh1 groups and STC1-sh1/ITGB6 OE groups)). Additionally, the results of cell migration and invasion assays showed that after transfection of the ITGB6 OE plasmid into the STC1-sh1 groups, the cell migration and invasion ability was up-regulated compared with that of the STC1-sh1 groups and was not significantly different compared with that of the NC groups (Supplementary Fig. 4D-F (Cell migration and invasion assays in Skov3-ip1 and Hey cell lines in the NC groups, STC1-sh1 groups and STC1-sh1/ITGB6 OE groups)). The results of ORO staining showed that there was greater accumulation of TGs (red arrow) in the cytoplasm in the STC1-sh1/ITGB6 OE groups than in that of the STC1-sh1 groups (Supplementary Fig. 4G (ORO staining in Skov3-ip1 and Hey cell lines in the NC groups, STC1-sh1 groups and STC1-sh1/ITGB6 OE groups (red arrow: TGs))). These results suggested that the decreases in cell proliferation and migration induced by STC1 knockdown were rescued by transfection of the ITGB6 OE plasmid into two OC cell lines, which proved that ITGB6 had feedback regulations to STC1. In conclusion, we concluded that STC1 directly bound to ITGB6 to activate the PI3K signaling pathway in vitro.

Then, we explored the relationship between ITGB6 expression and lipid metabolism. We conducted untargeted relative quantitative lipidomic assays using ITGB6-sh1 and ITGB6-sh2 Hey cells and their corresponding controls. Systematic lipidomic changes occurring in different groups were then assessed by PCA (Fig. 4G). We identified 11 significantly changed lipids from 5 lipid classes in ITGB6-sh1/sh2 cells compared to control cells (Supplementary Fig. 4H (11 significantly changed lipids from 5 lipid classes were identified in the ITGB6-sh1/sh2 groups compared with the NC group)). To summarize the individual lipid species significantly up- or down- regulated in control cells compared to ITGB6-sh1 and ITGB6-sh2 cells, volcano plots showed these lipids were generated and were presented in Fig. 4H. Lipids with a P value < 0.05 and a fold change (FC) > 1.5 or < 0.67 were indicated in blue or red, respectively. The results of the correlation analysis of the 5 lipid classes were presented in Fig. 4I. Metabolomics analyses were next performed. Significant differences in the variables among the control group, ITGB6-sh1 group and ITGB6-sh2 group were depicted as a heat map in Supplementary Fig. 4I (A heat map of significant differences in the variables among the NC group, ITGB6-sh1 and ITGB6-sh2 groups of Hey cells), and were ranked by ANOVA. Each row represented an individual lipid, and each column represented an individual sample. Interestingly, correlation analysis among the 11 lipids revealed 2 distinct clusters. In the ITGB6-sh1 and ITGB6-sh2 groups, some PCs were down-regulated, while a small number of phosphatidylethanol amines (PE), sphingomyelin (SM) and phosphatidylserine (PS) lipids were up-regulated. The results of untargeted relative quantitative lipidomic assays for the ITGB6-sh1/sh2 groups were similar to those for the STC1-sh1/sh2 groups. Therefore, we concluded that ITGB6 was participated in lipid metabolism in vitro.

In addition, we knocked down ITGB6 (ITGB6-sh1 and ITGB6-sh2) in two cell lines. Then, we conducted WB experiments and found that the protein level of ITGB6 was significantly decreased in the ITGB6-sh1 and ITGB6-sh2 groups in Skov3-ip1 and Hey cell lines. Moreover, the stable knockdown of ITGB6 decreased the expression level of phosphorylated PI3K, FAK and AKT (Fig. 4J). Therefore, we concluded that ITGB6 activated the PI3K signaling pathway. In all, we demonstrated that STC1 bound to ITGB6 to activate the PI3K signaling pathway, participating in lipid metabolism in vitro.

### STC1 is directly regulated by FOXC2 to activate the PI3K signaling pathway, participating in lipid metabolism in vitro

**Fig. 5:**
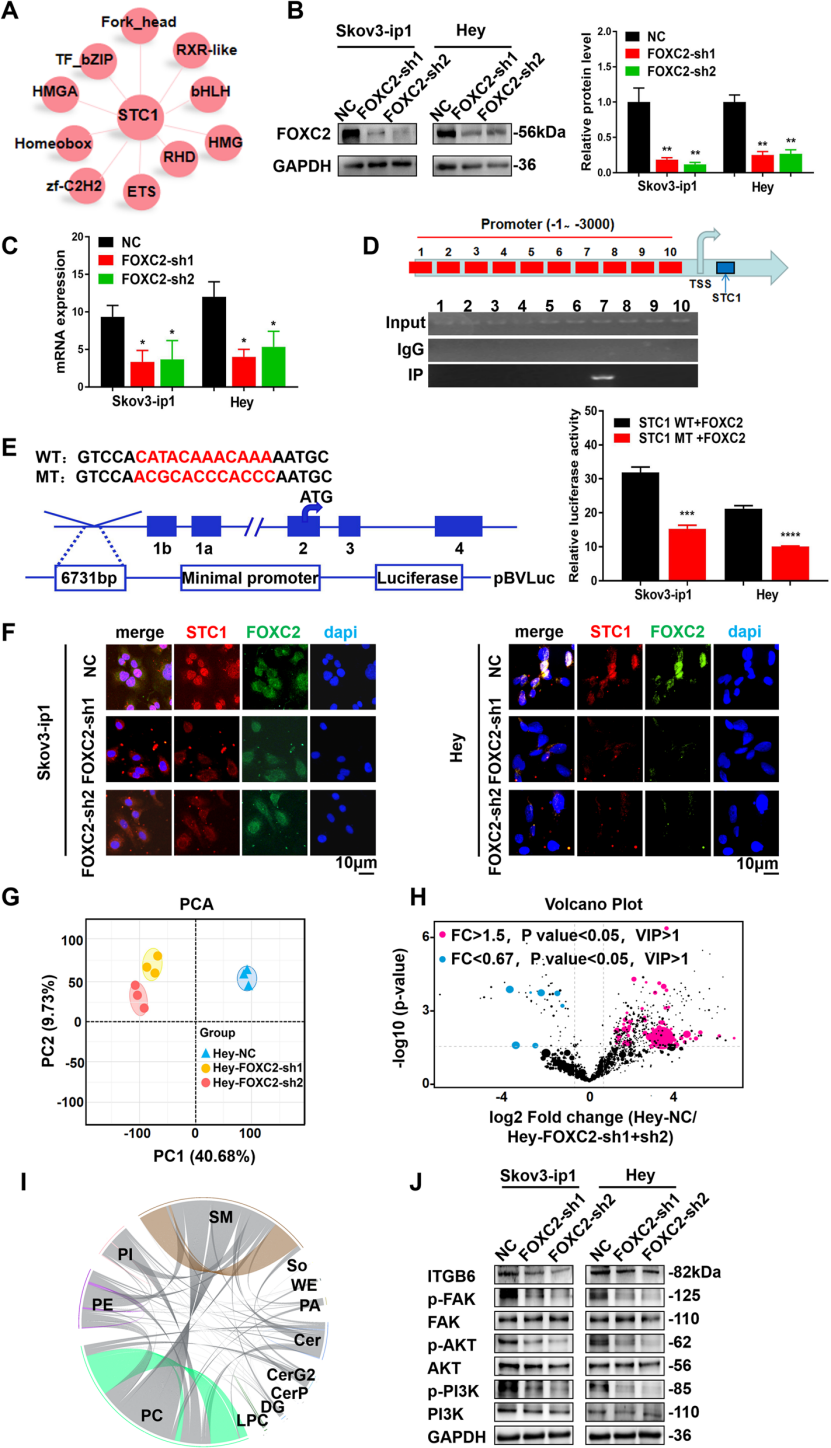
STC1 is directly regulated by FOXC2 to activate the PI3K signaling pathway, participating in lipid metabolism in vitro. **A** Ten transcription factor families that may be related to STC1 were identified by RNA sequencing. **B-C** Stable knockdown of FOXC2 expression in Skov3-ip1 and Hey cells was confirmed by WB and real‐time PCR assays. **D** ChIP results of the binding of FOXC2 to the promoter of STC1. **E** The luciferase reporter assay showed that co-transfection of the STC1 MT plasmid with the FOXC2 plasmid significantly decreased luciferase activity. **F** IF analysis showed the co-localization of FOXC2 and STC1 in Skov3-ip1 and Hey cell lines. **G** PCA of data from untargeted relative quantitative lipidomic analysis. **H** Volcano plot of individual lipid species significantly up- or down- regulated in the NC group versus the FOXC2-sh1 and FOXC2-sh2 groups in Hey cell lines (lipids with a *P* value < 0.05 and a fold change (FC) > 1.5 or < 0.67 were indicated in blue or red, respectively). **I** Correlation analysis of the 12 lipid classes. **J** Representative WB results of ITGB6 and proteins in the PI3K signaling pathway in Skov3-ip1 and Hey cell lines. (Data are shown as the mean ± SD values. Significance was calculated using Student’s t test. *, *P* < 0.05; **, *P* < 0.01; ***, *P* < 0.001; ****, *P* < 0.0001.)

Through RNA sequencing, we found that there were 10 transcription factor families that may be related to STC1 (Fig. 5A). We thus examined the potential proteins associated with STC1 via www.cbioportal.org and found that FOXC2, one of the closely related members of the Forkhead box (FOX) transcription factor family, was an upstream gene of STC1 (Supplementary Fig. 5A (FOXC2 has interaction with STC1)). The p-value was 4.909e^−5^. Then, we knocked down FOXC2 (FOXC2-sh1 and FOXC2-sh2) in two cell lines. The WB and qPCR results demonstrated that the protein expression and mRNA expression levels of FOXC2 were significantly down-regulated (Fig. 5B-C). Then, to determine the exact region within the FOXC2 promoter that STC1 binds, we performed ChIP assay with the Skov3-ip1 cell line. The experiments identified one STC1 binding site, which was close to the transcription start sites (TSS) (Fig. 5D). In addition, to further determine the binding site for STC1 in the promoter of FOXC2, we generated individual mutations in STC1 and repeated the luciferase reporter assay. The data showed that mutation of the binding site alone abrogated the luciferase activity (Fig. 5E). Then, we conducted IF analysis (Fig. 5F), and the data showed co-localization of FOXC2 and STC1. On the other hand, to further address the relationship between STC1 and FOXC2, we transfected the STC1 OE plasmid into FOXC2-sh1 groups of two cell lines (Supplementary Fig. 5B (The knockdown of FOXC2 expression level in the FOXC2-sh1 groups were rescued by STC1 OE plasmid in Skov3-ip1 and Hey cell lines)). We found that after transfection of the STC1 OE plasmid into the FOXC2-sh1 group, the protein expression level of FOXC2 was up-regulated accordingly in two OC cell lines, which demonstrated that STC1 interacted with FOXC2. Next, the results of cell proliferation assays showed that after transfection of the STC1 OE plasmid into the FOXC2-sh1 groups, the cell proliferation ability was up-regulated compared with that of the FOXC2-sh1 groups and was not significantly different compared with that of the NC groups (Supplementary Fig. 5C (Cell proliferation assays in Skov3-ip1 and Hey cell lines in the NC groups, FOXC2-sh1 groups and FOXC2-sh1/STC1 OE groups)). Additionally, the results of cell migration and invasion experiments showed that after transfection of the STC1 OE plasmid into the FOXC2-sh1 groups, the cell migration and invasion ability was increased compared with that of the FOXC2-sh1 groups and was not significantly different compared with that of the NC groups (Supplementary Fig. 5D-F (Cell migration and invasion in Skov3-ip1 and Hey cell lines in the NC groups, FOXC2-sh1 groups and FOXC2-sh1/STC1 OE groups)). These results suggested that the decreases in cell proliferation and migration ability induced by FOXC2 knockdown was rescued by transfection of the STC1 OE plasmid in two OC cell lines, which proved that STC1 had feedback regulations to FOXC2. In summary, these results further confirmed that STC1 was directly regulated by FOXC2 in vitro.

Then, we explored the relationship between FOXC2 expression and lipid metabolism. We conducted untargeted relative quantitative lipidomic assays using FOXC2-sh1 and FOXC2-sh2 Hey cells and their corresponding controls. Systematic lipidomic changes occurring in different groups were then assessed by PCA (Fig. 5G). We identified 87 significantly changed lipids from 12 lipid classes in FOXC2-sh1/sh2 cells compared to control cells (Supplementary Fig. 5G (87 significantly changed lipids from 12 lipid classes were identified in the FOXC2-sh1/sh2 groups compared with the NC group)). To summarize the individual lipid species significantly up- or down- regulated in control cells compared to FOXC2-sh1 and FOXC2-sh2 cells, volcano plots showed these lipids were generated and were presented in Fig. 5H. Lipids with a *P* value < 0.05 and a fold change (FC) > 1.5 or < 0.67 were indicated in blue or red, respectively. The results of the correlation analysis of the 12 lipid classes were presented in Fig. 5I. Metabolomics analyses were next performed. Significant differences in the variables among the control group, FOXC2-sh1 group and FOXC2-sh2 group were depicted as a heat map in Supplementary Fig. 5H (A heat map of significant differences in the variables among the NC group, FOXC2-sh1 groups and FOXC2-sh2 groups of Hey cells), and were ranked by ANOVA. Each row represented an individual lipid, and each column represented an individual sample. Interestingly, correlation values among the 12 lipids revealed 2 distinct clusters. Obviously, in the FOXC2-sh1 and FOXC2-sh2 groups, some PCs were down-regulated, while a large amount of PE, SM, PS and ceramide-G2 (CerG2) were up-regulated. The results of untargeted relative quantitative lipidomic assays for the FOXC2-sh1/sh2 groups were similar to those for the STC1-sh1/sh2 groups. Therefore, we concluded that FOXC2 was participated in lipid metabolism in vitro.

Furthermore, the WB assays indicated that the stable knockdown of FOXC2 decreased the expression levels of ITGB6, phosphorylated PI3K, FAK and AKT (Fig. 5J). Therefore, we concluded that FOXC2 regulated the protein expression level of ITGB6, and activated the PI3K signaling pathway. In all, we demonstrated that STC1 was directly regulated by FOXC2 to activate the PI3K signaling pathway, participating in lipid metabolism in vitro.

### STC1 promotes metastasis, lipid metabolism and DDP chemoresistance in vivo

**Fig. 6:**
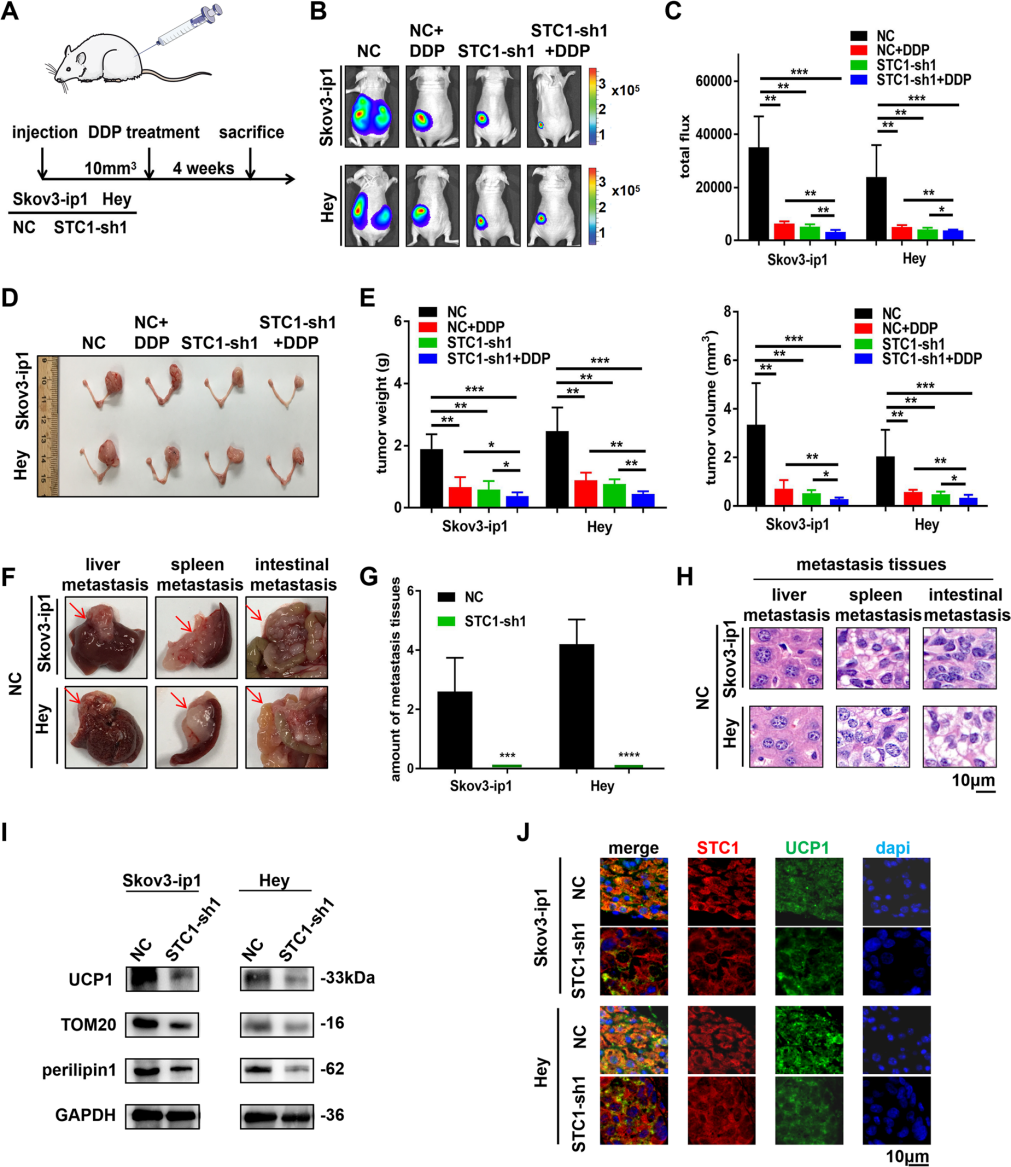
STC1 promotes metastasis, lipid metabolism and DDP chemoresistance in vivo. **A** The model of cell injection into nude mice. **B-C** Luciferase expression was detected in the mouse model. The NC group showed significantly higher flux than the NC + DDP group, STC1-sh1 group and STC1-sh1 + DDP group. **D** The NC group showed significantly larger tumors than the NC + DDP group, STC1-sh1 group and STC1-sh1 + DDP group. **E** The tumor weights and tumor volumes in the four groups. **F-G** The NC groups had metastatic tissues in the liver, spleen and intestine. **H** H&E staining of metastatic tissues (liver, spleen and intestine) in the NC groups. **I** Representative WB results of UCP1, TOM20 and perilipin1 in tissues from two mouse tumors generated by injection of the two cell lines. **J** IF analysis showed the relationship between STC1 and UCP1 in tissues from two mouse tumors generated by injection of the cell lines. (Data are shown as the mean ± SD values. Significance was calculated using Student’s t test. *, *P* < 0.05; **, *P* < 0.01; ***, *P* < 0.001; ****, *P* < 0.0001.)

To further confirm the role of STC1 in vivo, we established a nude mouse xenograft model (Fig. 6A). Tumor development occurred in 100% of mice with orthotopic injection into the ovaries, and longitudinal bioluminescence imaging was utilized to assess the tumor burden according to fluorescence (Fig. 6B). We determined the total flux of the tumors (Fig. 6C). The tumor tissues were shown in Fig. 6D. In addition, the tumor weight, tumor volume and ascites volume were measured (Fig. 6E and Supplementary Fig. 6A (The NC groups showed significantly more ascites than the STC1-sh1 groups)). We found that all of these parameters showed significant differences in the NC + DDP group, STC1-sh1 group and STC1-sh1 + DDP group compared with the NC group. There was a significant difference between the single treatment (NC + DDP and STC1-sh1) groups and the combined treatment (STC1-sh1 + DDP) groups. In the NC group, tumor metastases were found in the liver, spleen, peritoneum and colon, while we did not find metastases in the STC1-sh1 group (Fig. 6F-G). H&E staining of metastatic tissues were shown in Fig. 6H. In addition, H&E staining of tumor tissues in the NC group, NC + DDP group, STC1-sh1 group and STC1-sh1 + DDP group was shown in Supplementary Fig. 6B (H&E staining of tumor tissues in four groups). The IHC results showed that STC1 expression was decreased in the STC1-sh1 groups compared with the NC group (Supplementary Fig. 6C (IHC assays of STC1 in OC tissues)). These data suggested that STC1 promoted metastasis and DDP chemoresistance in vivo.

Moreover, we further explored whether STC1 could promote lipid metabolism in vivo. We conducted blood tests focusing on the TG level of the mice. The results showed that the TG level in the NC groups was higher than that in the STC1-sh1 groups (Supplementary Fig. 6D (The NC groups showed higher level of blood TG compared with the STC1-sh1 groups)). Furthermore, the protein expression levels of UCP1, TOM20 and perilipin1 was decreased in the STC1-sh1 groups compared with the NC groups, as shown by WB analysis of mouse tumor tissues (Fig. 6I). IF analysis confirmed the down-regulation of UCP1, TOM20 and perilipin1 in STC1 knockdown tumor tissues (Fig. 6J and Supplementary Fig. 6E (IF assay detected the relationship between STC1, TOM20 and perilipin1 in two cell lines in mouse tumor tissues)). Thus, we demonstrated that STC1 promoted lipid metabolism by up-regulating lipid-related genes such as UCP1, TOM20 and perilipin1 in vivo.

In addition, the results of WB analysis demonstrated that the protein expression levels of members in the integrin family (ITGAV, ITGB6 and ITGB7) had decreased in the STC1-sh1 groups compared with the NC groups (Supplementary Fig. 6F (IHC assays of proteins in PI3K signaling pathway in mouse tumor tissues)). The IHC assays of PI3K, AKT and FAK in tumor tissues were showed in Supplementary Fig. 6G (STC1 influenced the protein expression levels of members in the integrin family in mouse tumor tissues by WB assays). Besides, the results of IF analysis showed co-localization of FOXC2 and STC1 in mouse tissues, as well as co-localization of STC1 and ITGB6 in mouse tissues (Supplementary Fig. 7A-B (IF assay detected the relationship of STC1 and FOXC2, STC1 and ITGB6 in mouse tumor tissues in two cell lines)). In summary, these results indicated that STC1 promoted metastasis, lipid metabolism and DDP chemoresistance in vivo, while the FOXC2/ITGB6 signaling axis and the PI3K signaling pathway may be involved in the process.

### Targeting STC1 and the FOXC2/ITGB6 signaling axis is related to DDP chemoresistance in vitro

**Fig. 7:**
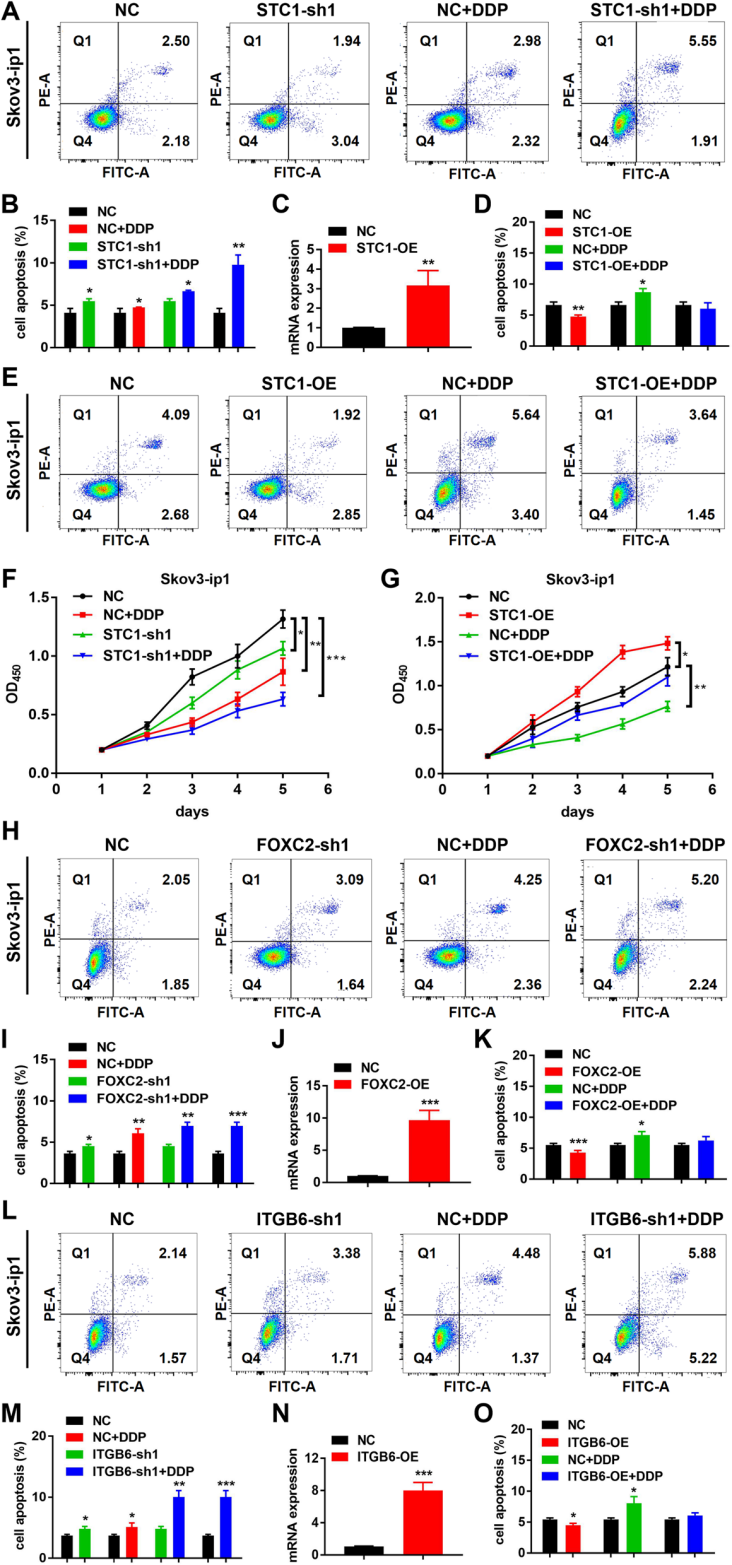
Targeting STC1 and the FOXC2/ITGB6 signaling axis is related to DDP chemoresistance in vitro. **A-B** Cell apoptosis results in the NC, NC + DDP, STC1-sh1 and STC1-sh1 + DDP groups in Skov3-ip1 cells. **C** The mRNA level of STC1-OE expression in Skov3-ip1 cells was confirmed by real‐time PCR. **D-E** Cell apoptosis results in the NC, STC1-OE, NC + DDP and STC1-OE + DDP groups in Skov3-ip1 cell lines. **F** The results of cell proliferation assays in the NC, NC + DDP, STC1-sh1 and STC1-sh1 + DDP groups in Skov3-ip1 cells. **G** The results of cell proliferation assays in the NC, STC1-OE, NC + DDP and STC1-OE + DDP groups in Skov3-ip1 cell lines. **H-I** Cell apoptosis results in the NC, NC + DDP, FOXC2-sh1 and FOXC2-sh1 + DDP groups in Skov3-ip1 cells. **J** The mRNA level of FOXC2-OE expression in Skov3-ip1 cells was confirmed by real‐time PCR. **K** Cell apoptosis results in the NC, FOXC2-OE, NC + DDP and FOXC2-OE + DDP groups in Skov3-ip1 cell lines. **L-M** Cell apoptosis results in the NC, NC + DDP, ITGB6-sh1 and ITGB6-sh1 + DDP groups in Skov3-ip1 cells. **N** The mRNA level of ITGB6-OE expression in Skov3-ip1 cells was confirmed by real‐time PCR. **O** Cell apoptosis results in the NC, ITGB6-OE, NC + DDP and ITGB6-OE + DDP groups in Skov3-ip1 cell lines. (Data are shown as the means ± SD. Significance was calculated using Student’s t test. *, *P* < 0.05, **, *P* < 0.01, ***, *P* < 0.001, ****, *P* < 0.0001.)

To explore whether STC1 could be a therapeutic target in OC, we conducted apoptosis assays and cell proliferation experiments in Skov3-ip1 cell lines. The results of apoptosis assays showed that the number of apoptotic cells was significantly increased in the STC1-sh1 group compared with the NC group. After treatment with DDP, the number of apoptotic cells was significantly increased in the NC + DDP group compared with the NC group. The number of apoptotic cells was further increased in the STC1-sh1 + DDP group compared with the NC group (Fig. 7A-B). These results indicated that knockdown of STC1 could have a synergistic effect with DDP in vitro. In addition, to further explore the correlation between the action of DDP and the expression of STC1, we also transfected the STC1 OE plasmid into Skov3-ip1 cells and established the STC1-OE cell lines (Fig. 7C). Then, we demonstrated that the OE of STC1 reversed the effect of DDP in apoptosis assays (Fig. 7D-E). Next, the results of cell proliferation assays showed that the ability of cell proliferation was significantly decreased in the STC1-sh1 group compared with the NC group. After treatment with DDP, the cell proliferation ability was also significantly decreased in the NC + DDP group compared with the NC group. Cell proliferation was further decreased in the STC1-sh1 + DDP group compared with the NC group (Fig. 7F). After that, we demonstrated that OE of STC1 reversed the effect of DDP in cell proliferation assays (Fig. 7G). These results indicated that STC1 promoted DDP chemoresistance in vitro.

Then, to explore whether FOXC2 could promoted DDP chemoresistance, we conducted apoptosis assays and cell proliferation experiments in Skov3-ip1 cell lines as well. The results of apoptosis assays showed that the number of apoptotic cells was significantly increased in the FOXC2-sh1 group compared with the NC group. After treatment with DDP, the number of apoptotic cells was significantly increased in the NC + DDP group compared with the NC group. The number of apoptotic cells was further increased in the FOXC2-sh1 + DDP group compared with the NC group (Fig. 7H-I). These results indicated that knockdown of FOXC2 could have a synergistic effect with DDP in vitro. In addition, to explore the correlation between the action of DDP and FOXC2, we also transfected the FOXC2 OE plasmid into Skov3-ip1 cells and established the FOXC2-OE cell lines (Fig. 7J). Then, we demonstrated that OE of FOXC2 reversed the effect of DDP in apoptosis assays (Fig. 7K and Supplementary Fig. 8A (Cell apoptosis results in the NC, FOXC2-OE, NC+DDP and FOXC2-OE+DDP groups in Skov3-ip1 cell lines)). Next, the results of cell proliferation assays showed that the ability of cell proliferation was significantly decreased in the FOXC2-sh1 group compared with the NC group. After treatment with DDP, the cell proliferation ability was also significantly decreased in the NC + DDP group compared with the NC group. Then, we found that cell proliferation was further decreased in the FOXC2-sh1 + DDP group compared with the NC group (Supplementary Fig. 8B (The results of cell proliferation assays in the NC, NC+DDP, FOXC2-sh1 and FOXC2-sh1+DDP groups in Skov3-ip1 cells)). After that, we demonstrated that OE of FOXC2 reversed the effect of DDP in cell proliferation assays (Supplementary Fig. 8C (The results of cell proliferation assays in the NC, FOXC2-OE, NC+DDP and FOXC2-OE+DDP groups in Skov3-ip1 cell lines)). These results indicated that FOXC2 promoted DDP chemoresistance in vitro.

Next, to explore whether ITGB6 could promoted DDP chemoresistance, we conducted apoptosis assays and cell proliferation experiments in Skov3-ip1 cell lines as well. The results of apoptosis assays showed that the number of apoptotic cells was significantly increased in the ITGB6-sh1 group compared with the NC group. After treatment with DDP, the number of apoptotic cells was significantly increased in the NC + DDP group compared with the NC group. The number of apoptotic cells was further increased in the ITGB6-sh1 + DDP group compared with the NC group (Fig. 7L-M). These results indicated that knockdown of ITGB6 could have a synergistic effect with DDP in vitro. In addition, to explore the correlation between the action of DDP and ITGB6, we also transfected the ITGB6 OE plasmid into Skov3-ip1 cells and established the ITGB6-OE cell lines (Fig. 7N). Then, we demonstrated that the OE of ITGB6 reversed the effect of DDP in apoptosis assays (Fig. 7O and Supplementary Fig. 8D (Cell apoptosis results in the NC, ITGB6-OE, NC+DDP and ITGB6-OE+DDP groups in Skov3-ip1cell lines)). Next, the results of cell proliferation assays showed that cell proliferation was significantly decreased in the ITGB6-sh1 group compared with the NC group. After treatment with DDP, the cell proliferation ability was also significantly decreased in the NC + DDP group compared with the NC group. Then, we found that cell proliferation was further decreased in the ITGB6-sh1 + DDP group compared with the NC group (Supplementary Fig. 8E (The results of cell proliferation assays in the NC, NC+DDP, ITGB6-sh1 and ITGB6-sh1+DDP groups in Skov3-ip1 cells)). After that, we demonstrated that OE of ITGB6 reversed the effect of DDP in cell proliferation assays (Supplementary Fig. 8F (The results of cell proliferation assays in the NC, ITGB6-OE, NC+DDP and ITGB6-OE+DDP groups in Skov3-ip1 cell lines)). These results indicated that ITGB6 promoted DDP chemoresistance in vitro. In all, we confirmed that targeting STC1 and the FOXC2/ITGB6 signaling axis was related to DDP chemoresistance in vitro.

### The expression of FOXC2, STC1 and ITGB6 is associated with poor survival in OC patients

**Fig. 8:**
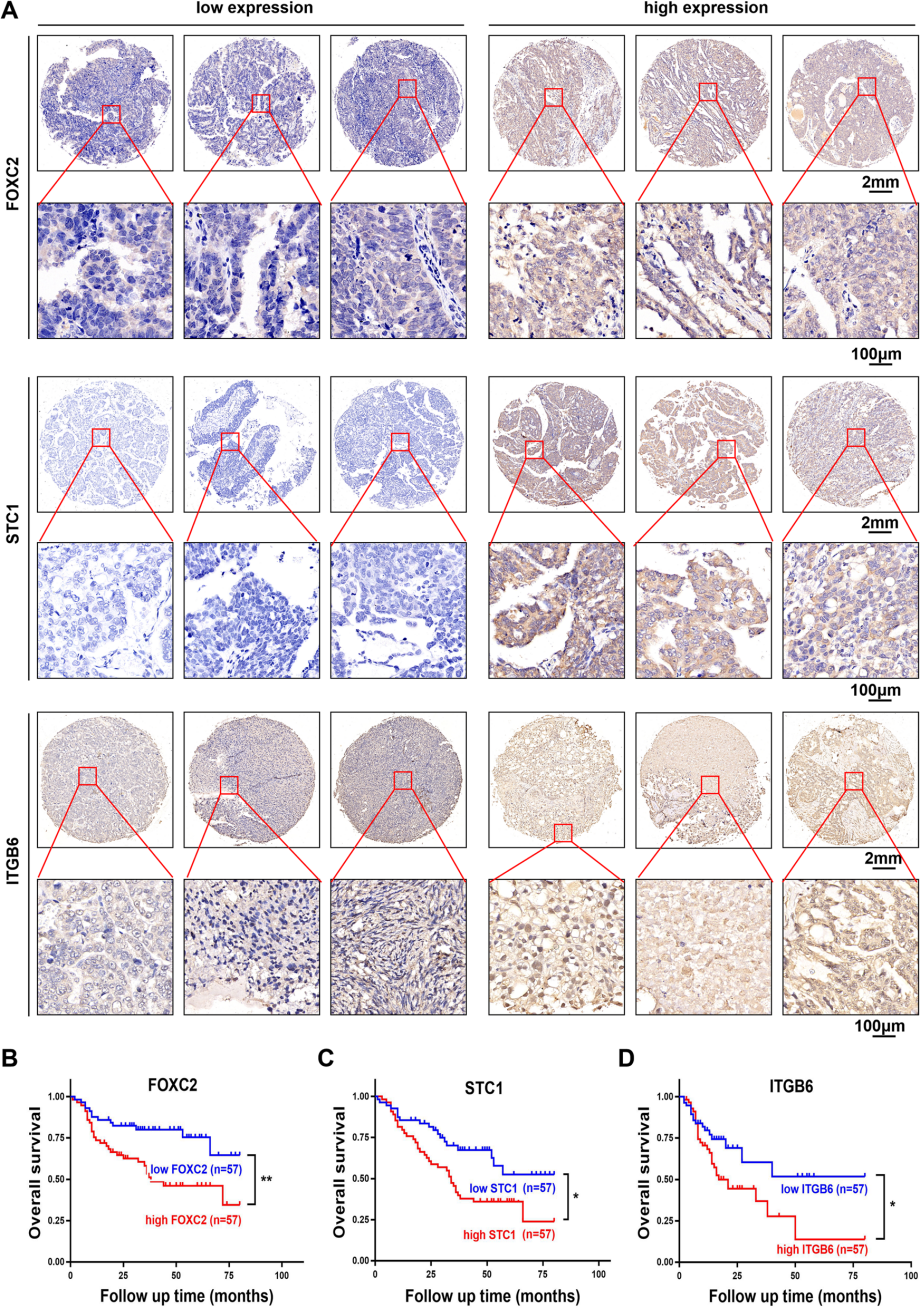
The expression of FOXC2, STC1 and ITGB6 is associated with poor survival in OC patients. **A** Representative images of IHC staining of FOXC2, STC1 and ITGB6 in OC tissues. **B-D** OS of OC patients with different expression levels of FOXC2, STC1 and ITGB6. (Data are shown as the mean ± SD values. Significance was calculated using Student’s t test. *, *P* < 0.05; **, *P* < 0.01; ***, *P* < 0.001; ****, *P* < 0.0001.)

**Fig. 9:**
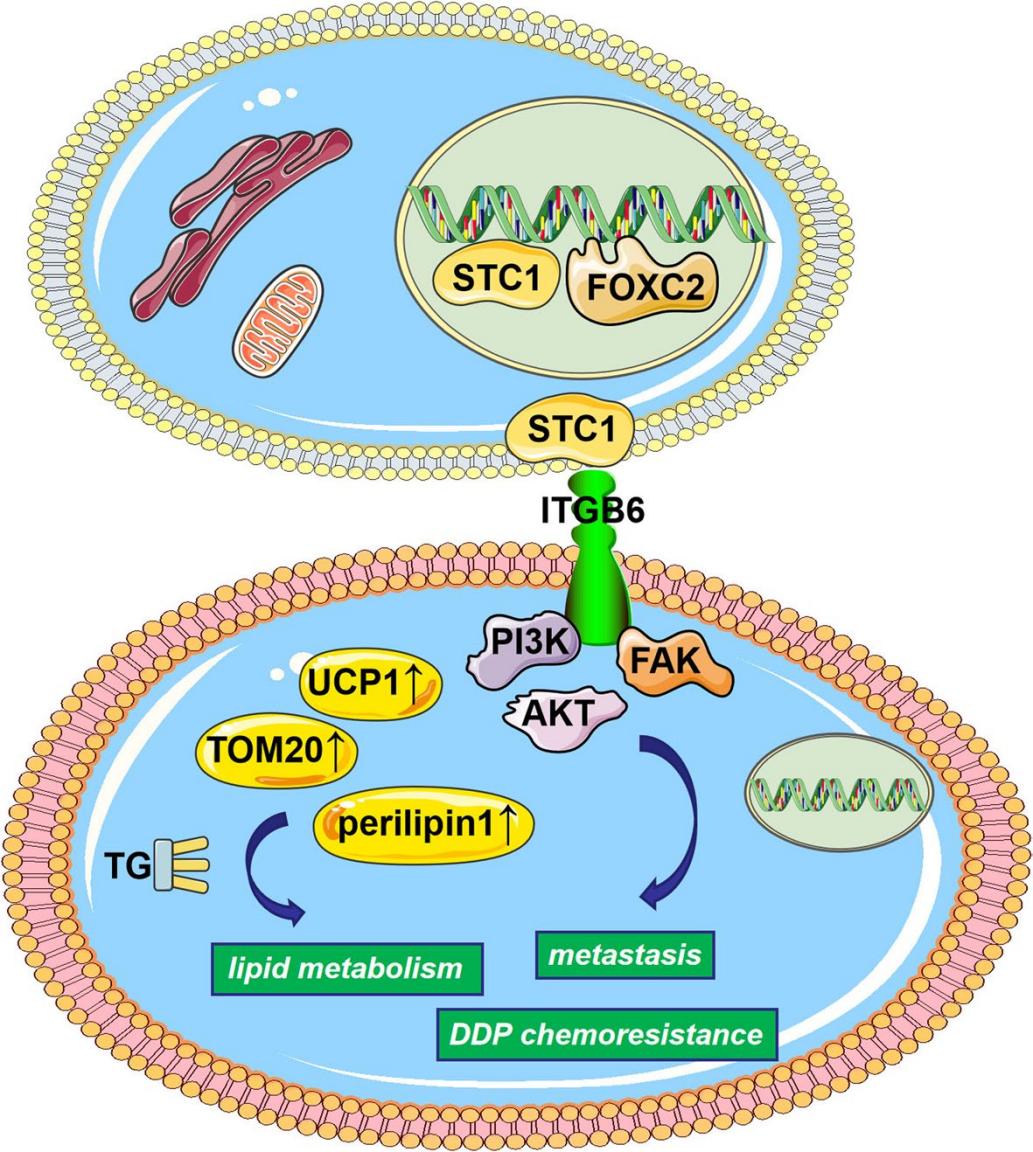
Schematic model showing the role of the FOXC2/STC1/ITGB6 signaling axis in the regulation of metastasis, lipid metabolism and DDP chemoresistance in OC cells

To demonstrate the clinical significance of FOXC2, STC1 and ITGB6 in OC, we estimated their expression levels through TMA analysis (Fig. 8A). High expression of FOXC2, STC1 and ITGB6 was associated with poor OS in OC patients (Fig. 8B-D). In addition, the H&E staining of the TMAs is shown in Supplementary Fig. 9A (H&E staining of the TMAs of FOXC2, STC1 and ITGB6 in OC tissues). In summary, we demonstrated that STC1 promoted tumor metastasis, lipid metabolism and DDP chemoresistance in OC cells. In the meantime, STC1 promoted lipid metabolism by up-regulating lipid-related genes such as UCP1, TMO20 and perilipin1. Moreover, FOXC2 acted as a transcription factor by binding to the promoter region of STC1, and then STC1 interacted with ITGB6 to influence genes involved in the PI3K signaling pathway, such as PI3K, FAK and AKT (Fig. 9).

## Discussion

STC1 plays critical roles in tumor progression. However, the mechanism of STC1 in metastasis, lipid metabolism and DDP chemoresistance in OC remains elusive. In this study, we indicated that STC1 promoted metastasis, lipid metabolism and DDP chemoresistance via the FOXC2/ITGB6 signaling axis in OC. In the meantime, STC1 promoted lipid metabolism by up-regulating lipid-related genes such as UCP1, TOM20 and perilipin1. Moreover, targeting STC1 and the FOXC2/ITGB6 signaling axis would be a new target for OC patients, especially those who have developed chemoresistance to DDP.

A novel finding of our analyses is that STC1 promoted metastasis and lipid metabolism in OC. The results of single-cell sequencing and IHC assays verified that STC1 expression was significantly enhanced in OC tissues compared with para-carcinoma tissues, and it was further up-regulated in peritoneal metastasis tissues compared with OC tissues. Through further analysis, we concluded that STC1 was closely associated with OC metastasis and the metastasis was relate to lipid metabolism. Due to the almost symbiotic relationship between OC and fat-containing cells in the omentum, researchers have shown great interest in advancing the understanding of how lipid metabolism fuels peritoneal dissemination and supplies energy for tumor progression in OC [41, 42]. Some studies have shown that OC cells utilize various strategies to boost lipid uptake to fulfil the high energy requirement for cell growth [6, 43]. On the other hand, this increase was hypothesized to strongly support the importance of increased lipid uptake in promoting tumor metastasis in OC [44, 45]. We are the first to find that STC1 promoted the metastasis of OC cells and the metastasis was related to lipid metabolism. In vitro, we demonstrated that STC1 promoted OC cells metastasis through migration and invasion assays and wound healing experiments. Furthermore, our results of untargeted relative quantitative lipidomic assays indicated that compared with control cells, STC1 knockdown cancer cells contained decreased amounts of TGs (such as 16:0 and 18:0) and increased amounts of PCs (such as 36:0) and Cers (such as d18:1). TGs are related to metabolic syndromes, which are obviously related to increased OC risk [46, 47] and lead to poor prognosis [48] in OC patients. Studies have shown that fatty acids derived from TGs can be used for energy and membrane synthesis in proliferating cells [49]. In other studies, TGs were associated with increased risks of OC [50] and had a statistically significant association with advanced tumor stage, low differentiation, positive lymph node metastasis and a poor prognosis [46]. Moreover, in our study, we found that there were greater numbers of mitochondria in the control cells than in the STC1-sh1 and STC1-sh2 cells in TEM results. In addition, the results of ORO staining in OC cells showed that large amounts of TGs accumulated in the cytoplasm in the cells of the control group. Thus, we concluded that STC1 promoted tumor metastasis and lipid metabolism in vitro. In the meantime, TGs may be universally involved in this process.

Interestingly, our results showed that STC1 promoted lipid metabolism in OC cells by up-regulated lipid-related genes such as TOM20, UCP1 and perilipin1. First, we evaluated overall mitochondrial function by measuring the OCR and ECAR in the two cell lines. The glycolysis level and reserve capacity in STC1-sh1 and STC1-sh2 cells were dramatically decreased compared with those in control cells. The results indicated that STC1 promoted lipid metabolism in OC cells. Then, we conducted WB analysis and IF analysis. The results showed that the expression levels of UCP1, TOM20 and perilipin1 were down-regulated in STC1-sh1 and STC1-sh2 cells compared with those in control cells. Studies have shown that UCP1 is the most important marker of brown adipose tissue and it consume lipids to generate heat [51, 52]. TOM20 is one of the mitochondrial signal-anchored proteins, while abnormal TOM20 protein expression may be a sign of mitophagy [53]. Moreover, perilipin1 plays an important part in regulating lipid droplet formation, maintenance, and degradation[54, 55]. We are the first to demonstrate that STC1 promoted lipid metabolism by up-regulated lipid-related genes such as TOM20, UCP1 and perilipin1 in vitro. Overall, these findings revealed a novel mechanism underlying the promotive effect of STC1 on the metastasis and lipid metabolism in OC.

Mechanically, we indicated that STC1 was regulated by FOXC2 and then bound to ITGB6 to activate the PI3K signaling pathway, participating in lipid metabolism in vitro. Earlier study demonstrated that STC1 plays a critical role in tumor progression [27]. In the meantime, research showed that FOXC2 played a core role in cancer, as it was often overexpressed in OC [56]. While in our study, we first demonstrated that STC1 is regulated by the transcription factor FOXC2. We determined the exact region within the FOXC2 promoter that STC1 binds. In addition, the luciferase reporter assay was conducted to further determine the binding site for STC1 in the promoter of FOXC2. Our results of IF analysis showed co-localization of FOXC2 and STC1. Thus, we confirmed that STC1 was regulated by FOXC2 in vitro. Furthermore, we are also the first to identify ITGB6 as a previously unrecognized target of STC1 in OC cells. The results of IP assay and mass spectrometry analyses showed that STC1 was related to ITGB6. After that, the results of FRET-FLIM further demonstrated the direct interaction between STC1 and ITGB6. Then, we conducted co-IP assays, which showed that STC1 directly bound to ITGB6. Therefore, we concluded that STC1 bound to ITGB6 in OC cells. Simultaneously, our results demonstrated that the FOXC2/ITGB6 signaling axis was participated in lipid metabolism as well, which indicated that STC1 promoted lipid metabolism via the FOXC2/ITGB6 signaling axis in vitro.

Besides, we also found that STC1 bound to ITGB6 to activate the PI3K signaling pathway. The PI3K signaling pathway has long been a focus of interest in cancer due to its role in cancer cell proliferation, migration and metabolism. It is also related to lipid metabolism in many cancers such as gastric cancer [57], hepatocellular carcinoma [58] and breast cancer [59]. Recent reports have showed that the activation of the PI3K signaling pathway is linked to lipid synthesis [43] and lipid droplet accumulation [60] in OC. In our study, we proved that FOXC2, STC1 and ITGB6 could activate the PI3K signaling pathway in OC cells and that this process may be related to lipid metabolism. Thus, this novel FOXC2/STC1/ITGB6 signaling axis is a critical contributor to metastasis and lipid metabolism in OC cells.

In vivo, we found that STC1 promoted metastasis, lipid metabolism and DDP chemoresistance as well. We also demonstrated that STC1 promoted lipid metabolism by up-regulating lipid-related genes such as TOM20, UCP1 and perilipin1 in vivo. First of all, we established a nude mouse xenograft model and demonstrated that STC1 promoted metastasis of OC in vivo. Simultaneously, we found that STC1 promoted DDP chemoresistance in vivo. We discovered that the tumors were smaller in the STC1-KD group than in the control group. In STC1-KD mice that received DDP injection, the tumors were even smaller than those in the STC1-KD group. Thus, a unique feature of STC1 is of its ability to inhibit the chemotherapeutic effect of DDP in vivo. Next, WB analysis and IF analysis showed that the expression levels of UCP1, TOM20 and perilipin1 were down-regulated in STC1-sh1 cells of mice tissues compared with those in control cells. The results implied that STC1 promoted lipid metabolism of OC by up-regulating lipid-related genes such as UCP1, TOM20 and perilipin1 in vivo. Taken together, we concluded that STC1 promoted metastasis, lipid metabolism and DDP chemoresistance in vivo.

Lastly, we demonstrated that targeting STC1 and the FOXC2/ITGB6 signaling axis was related to DDP chemoresistance in vitro. To explore whether targeting STC1 and the FOXC2/ITGB6 signaling axis could be a therapeutic target in OC, we conducted apoptosis assays and cell proliferation experiments in Skov3-ip1 cell lines. We found that the number of apoptotic cells was significantly increased in the NC + DDP group compared with the NC group. The number of apoptotic cells was further increased in the STC1-sh1 + DDP group compared with the NC group. After that, we demonstrated that the OE of STC1 reversed the effect of DDP in apoptosis assays and cell proliferation assays. Thus, we confirmed that STC1 promoted DDP chemoresistance in vitro*.* Through apoptosis assays and cell proliferation experiments, we also verified that targeting the FOXC2/ITGB6 signaling axis was related to DDP chemoresistance in vitro. In brief, targeting STC1 and the FOXC2/ITGB6 signaling axis could be a novel therapeutic target in OC patients. Thus, it is worth further investigation.

## Conclusions

In our study, we indicated that STC1 is up-regulated in OC. Patients with a higher STC1 expression have a worse prognosis. STC1 promotes metastasis, lipid metabolism and DDP chemoresistance in OC. Simultaneously, STC1 promotes lipid metabolism by up-regulating lipid-related genes such as UCP1, TOM20 and perilipin1. Mechanistically, STC1 is regulated by FOXC2 and then binds to ITGB6 to activate the PI3K signaling pathway. Targeting STC1 and the FOXC2/ITGB6 signaling axis is related to DDP chemoresistance in OC cells. These results indicate that STC1 is a valuable prognostic biomarker and a promising target for anti-cancer therapy in OC, especially for those who have developed chemoresistance to DDP. Thus, STC1 could be a potential novel target to guide personalized therapy for OC patients. Targeting FOXC2/STC1/ITGB6 axis may influence the therapeutic effect of DDP in OC patients, and it is worth further consideration.

## Accession numbers

RNA sequencing data are available in the genomics data repository GEO under reference Series: GSE185833.

## Supplementary Information

**Additional file 1. **

## Data Availability

The data that support the findings of this study are available from the corresponding author upon reasonable request.

## Abbreviations

STC1: Stanniocalcin 1
OC: Ovarian cancer
ITGB6: Integrin β6
FOXC2: Forkhead box C2
DDP: Cisplatin
GSEA: Gene set enrichment analysis
TMA: Tissue microarray
IHC: Immunohistochemical
PBS: Phosphate buffer saline
ChIP: Chromatin Immunoprecipitation
OCR: Oxygen consumption rate
ECAR: Extracellular acidification rate
WB: Western blotting
OS: Overall survival
EOC: Epithelial ovarian cancer
