# Supplementary material for: Stanniocalcin 1 promotes metastasis, lipid metabolism and cisplatin chemoresistance via the FOXC2/ITGB6 signaling axis in ovarian cancer
Source: J Exp Clin Cancer Res. 2022 Apr 7;41:129. doi: 10.1186/s13046-022-02315-3 (PMC8988421; doi:10.1186/s13046-022-02315-3)
Supplement: Supplementary file 1 — Additional file 1. [file 13046_2022_2315_MOESM1_ESM.pdf]

Supplementary Table S1. The primary antibodies

Primary antibodies	Company (NO.)
STC1	Abcam (ab229477); Abcam (ab124891); Santa Cruz Biotechnology (sc-293435) ; Proteintech(20621-1-AP)
FOXC2	Abcam (ab5060); Abcam (ab245510); Proteintech (23066-1-AP); R&D (AF6989-SP)
ITGB6	Abcam (ab187155); Proteintech (19695-1-AP)
GAPDH	Proteintech (10494-1-AP)
PI3K	Proteintech (13329-1-AP)
FAK	Proteintech (12636-1-AP)
AKT	Proteintech (10176-2-AP)
ITGAV	Proteintech (27096-1-AP)
ITGB7	Abclonal (A5873)
UCP1	Proteintech (23673-1-AP); Beyotime (AF8292)
TOM20	Proteintech (11802-1-AP)
perilipin1	Abcam (ab172907)
p-FAK	CST (8556s)
p-AKT	Proteintech (86444-1-Ig)
p-PI3K	CST (17366s)

Supplementary Table S2. The primer sequences of qRT-PCR assay

Gene	Number	Primer
STC1	F	GTGGCGGCTCAAAACTCAG
	R	GTGGAGCACCTCCGAATGG
FOXC2	F	CCTCCTGGTATCTCAACCACA
	R	GAGGGTCGAGTTCTCAATCCC
ITGB6	F	TCCATCTGGAGTTGGCGAAAG
	R	TCTGTCTGCCTACACTGAGAG
GAPDH	F	GGAGCGAGATCCCTCCAAAAT
	R	GGCTGTTGTCATACTTCTCATGG

Supplementary Table S3. Association of STC1 expression with clinicopathological characteristics in 114 patients of EOC

	No. of patients (n = 114)	STC1 expression Low no. (%)	High no. (%)	P value
Characteristics				
Age (years)				> 0.05
<55	53	25(47.17%)	28(52.83%)	
≧55	61	24(46.15%)	37(53.85%)	
FIGO stage				< 0.05
I/II	42	38(90.48%)	4(9.52%)	
III/IV	72	19(26.39%)	53(73.61%)	
Grade				
1	34	30(88.24%)	4(11.76%)	
2	30	14(46.67%)	16(53.33%)	
3	50	11(22%)	39(78%)	
		Grade 2–3 versus 1		< 0.05
Lymph node				> 0.05
Positive	58	21(36.21%)	37(63.79%)	
Negative	56	32(57.14%)	24(42.86%)	

Figure S1

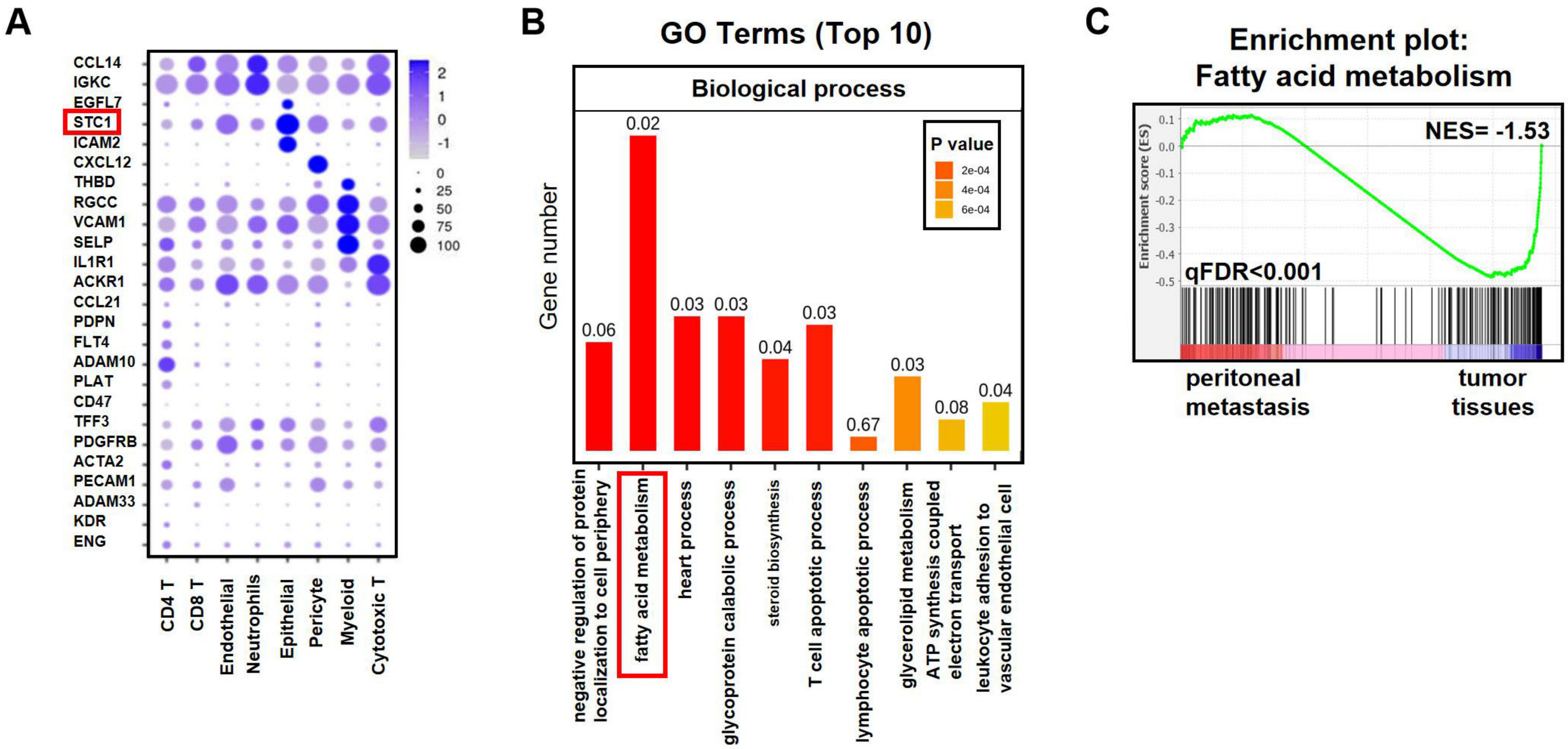
A
CCL14
IGKC
EGFL7
STC1
ICAM2
CXCL12
THBD
RGCC
VCAM1
SELP
IL1R1
ACKR1
CCL21
PDPN
FLT4
ADAM10
PLAT
CD47
TFF3
PDGFRB
ACTA2
PECAM1
ADAM33
KDR
ENG
2
1
0
-1
0
25
50
75
100
CD4 T
CD8 T
Endothelial
Neutrophils
Epithelial
Pericyte
Myeloid
Cytotoxic T
B
GO Terms (Top 10)
Biological process
Gene number
P value
2e-04
4e-04
6e-04
0.06
0.02
0.03
0.03
0.04
0.03
0.67
0.03
0.08
0.04
negative regulation of protein localization to cell periphery
fatty acid metabolism
heart process
glycoprotein calabolic process
steroid biosynthesis
T cell apoptotic process
lymphocyte apoptotic process
glycerolipid metabolism
ATP synthesis coupled electron transport
leukocyte adhesion to vascular endothelial cell
C
Enrichment plot:
Fatty acid metabolism
NES= -1.53
qFDR<0.001
Enrichment score (ES)
0.1
0.0
-0.1
-0.2
-0.3
-0.4
-0.5
peritoneal metastasis
tumor tissues

Figure S2

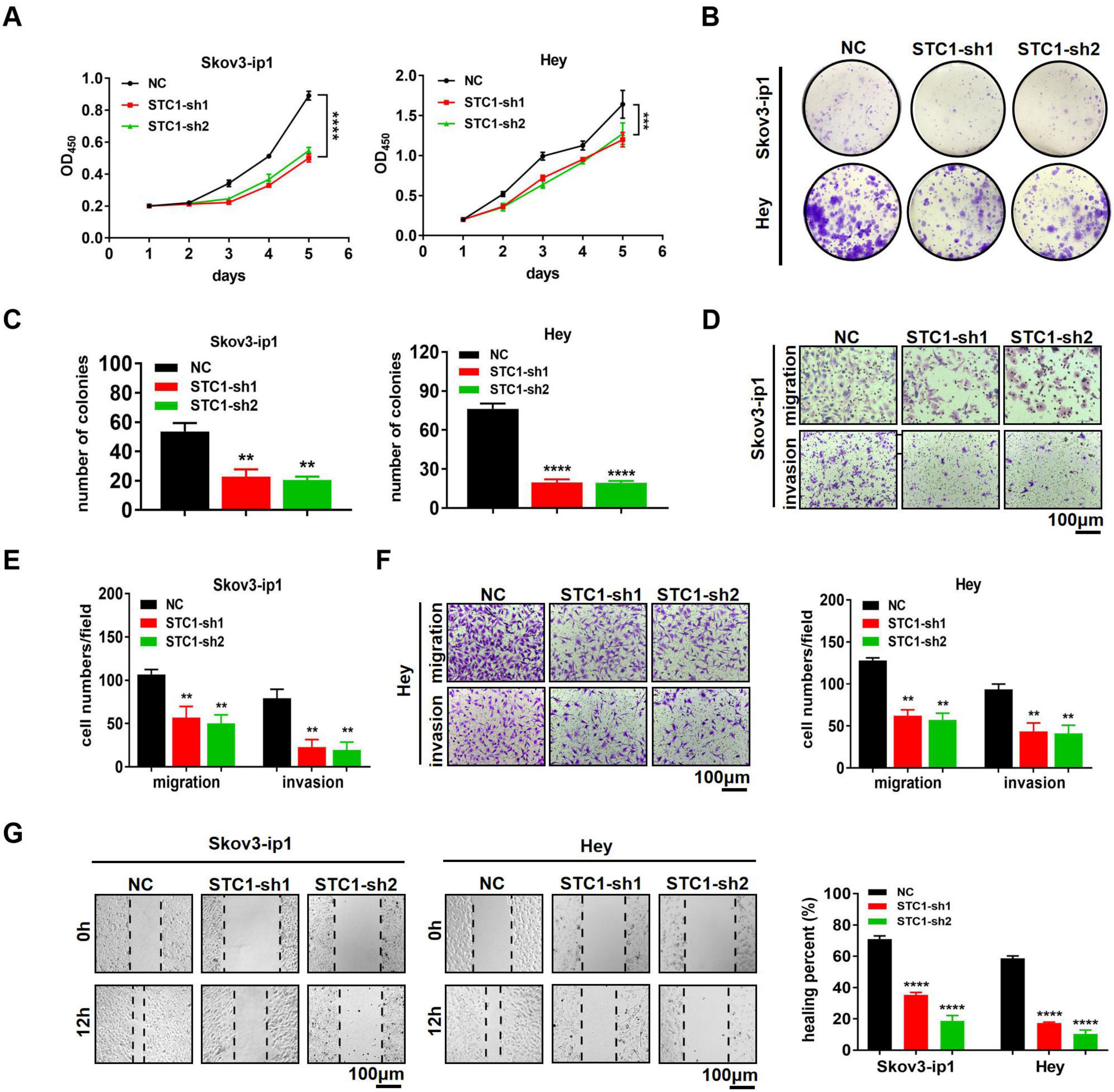

A
Skov3-ip1
NC
STC1-sh1
STC1-sh2
OD450
days
****
Hey
***
B
NC
STC1-sh1
STC1-sh2
Skov3-ip1
Hey
C
Skov3-ip1
number of colonies
**
Hey
****
D
migration
invasion
100μm
E
Skov3-ip1
cell numbers/field
migration
invasion
F
Hey
G
0h
12h
healing percent (%)

Figure S3

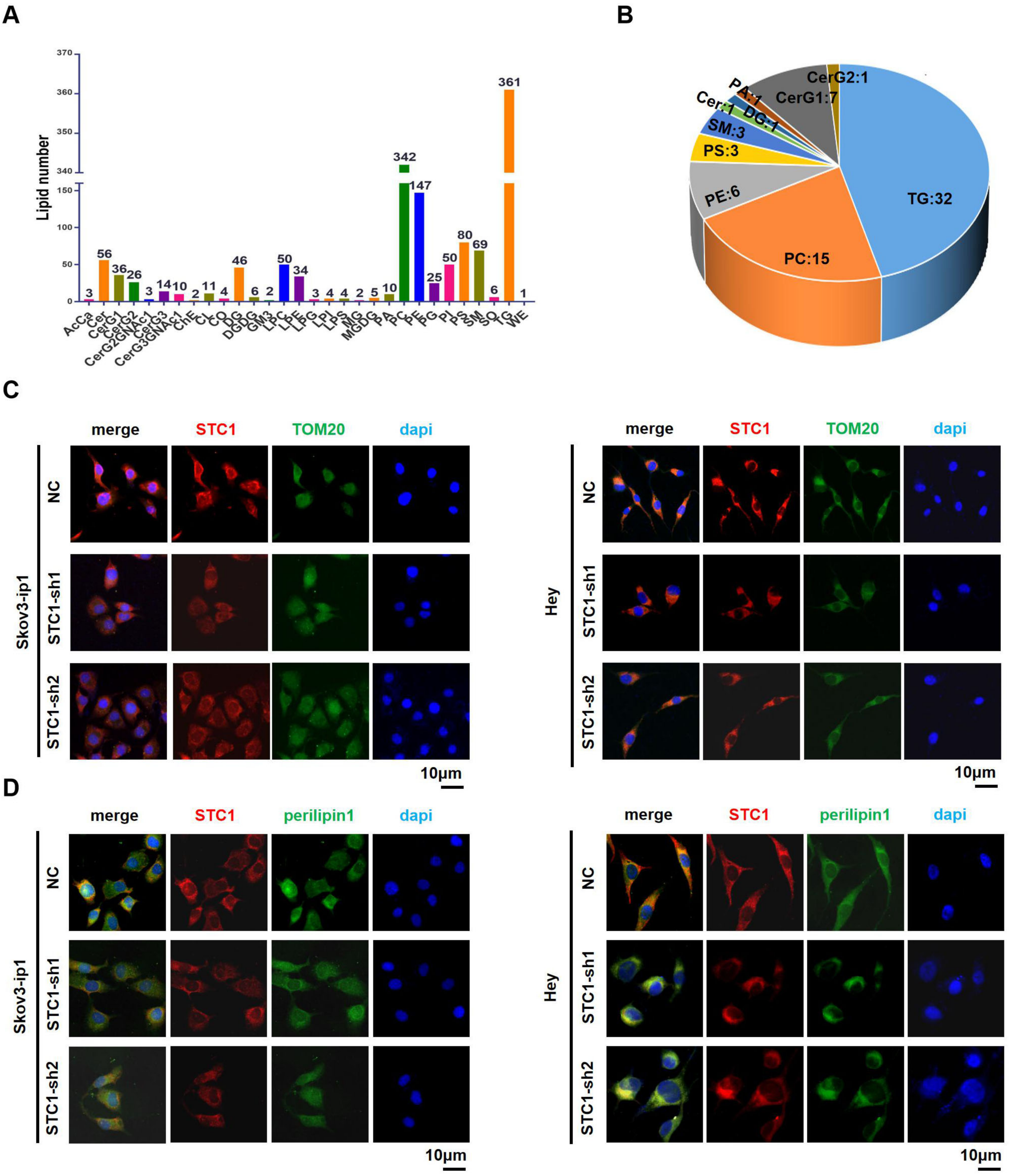
A
B
Lipid number
370
360
350
340
150
100
50
0
3
56
36
26
3
14
10
2
11
4
46
6
2
50
34
3
4
4
2
5
10
342
147
25
50
80
69
6
361
1
AcCa
Cer
CerG1
CerG2
CerG2GNAc1
CerG3
CerG3GNAc1
ChE
CL
CO
DG
DGDG
GM3
LPC
LPE
LPG
LPI
LPS
MG
MGDG
PA
PC
PE
PG
PI
PS
SM
SO
TG
WE
CerG2:1
PA:1
CerG1:7
Cer:1
DG:1
SM:3
PS:3
PE:6
TG:32
PC:15
C
merge
STC1
TOM20
dapi
Skov3-ip1
NC
STC1-sh1
STC1-sh2
10μm
Hey
NC
STC1-sh1
STC1-sh2
10μm
D
merge
STC1
perilipin1
dapi
Skov3-ip1
NC
STC1-sh1
STC1-sh2
10μm
Hey
NC
STC1-sh1
STC1-sh2
10μm

Figure S4

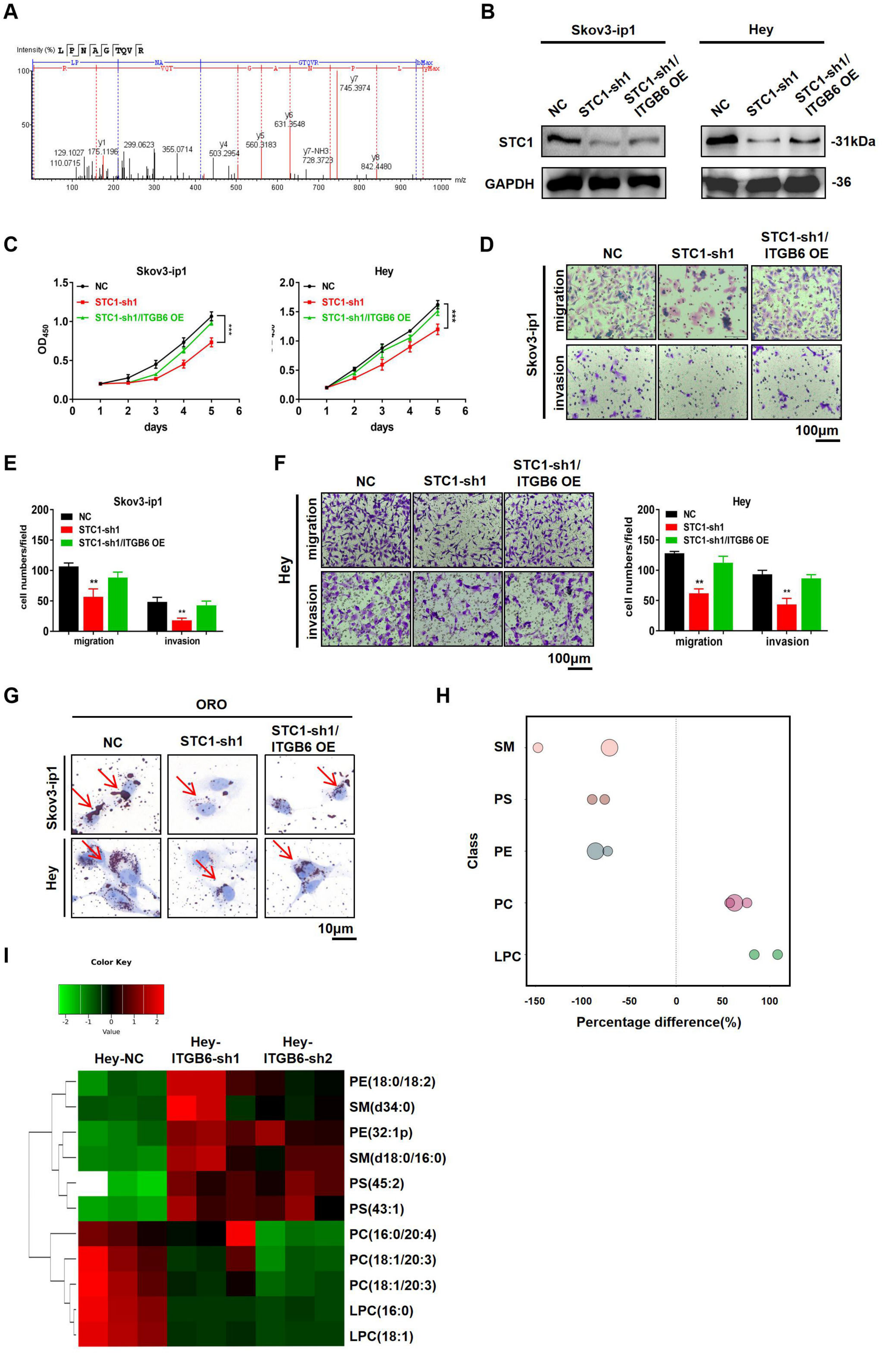

A
Intensity (%) L P N A G TQV R
LP
NA
GTQVR
bMax
R
VQT
G
A
N
P
L
yMax
y7
745.3974
y6
631.3548
y5
560.3183
y4
503.2954
y1
175.1196
129.1027
110.0715
299.0623
355.0714
y7-NH3
728.3723
y8
842.4480
m/z
B
Skov3-ip1
Hey
NC
STC1-sh1
STC1-sh1/ ITGB6 OE
STC1
GAPDH
-31kDa
-36
C
Skov3-ip1
Hey
NC
STC1-sh1
STC1-sh1/ITGB6 OE
OD450
***
days
D
NC
STC1-sh1
STC1-sh1/ ITGB6 OE
Skov3-ip1
migration
invasion
100µm
E
Skov3-ip1
NC
STC1-sh1
STC1-sh1/ITGB6 OE
cell numbers/field
**
migration
invasion
F
NC
STC1-sh1
STC1-sh1/ ITGB6 OE
Hey
migration
invasion
100µm
Hey
NC
STC1-sh1
STC1-sh1/ITGB6 OE
cell numbers/field
**
migration
invasion
G
ORO
NC
STC1-sh1
STC1-sh1/ ITGB6 OE
Skov3-ip1
Hey
10µm
H
SM
PS
PE
PC
LPC
Class
Percentage difference(%)
I
Color Key
Value
Hey-NC
Hey- ITGB6-sh1
Hey- ITGB6-sh2
PE(18:0/18:2)
SM(d34:0)
PE(32:1p)
SM(d18:0/16:0)
PS(45:2)
PS(43:1)
PC(16:0/20:4)
PC(18:1/20:3)
PC(18:1/20:3)
LPC(16:0)
LPC(18:1)

Figure S5

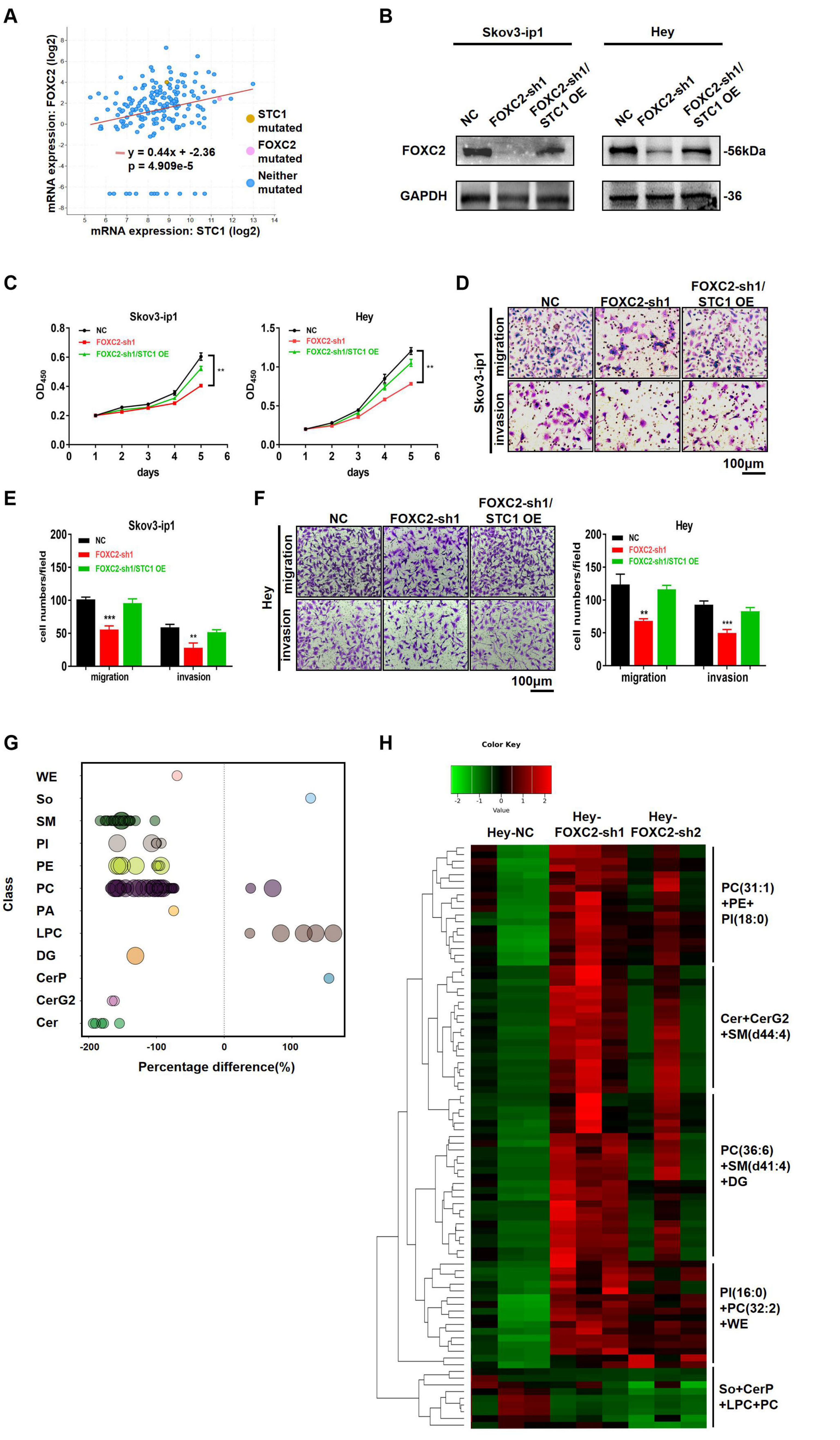

A
mRNA expression: FOXC2 (log2)
mRNA expression: STC1 (log2)
y = 0.44x + -2.36
p = 4.909e-5
STC1 mutated
FOXC2 mutated
Neither mutated
B
Skov3-ip1
Hey
NC
FOXC2-sh1
FOXC2-sh1/ STC1 OE
FOXC2
GAPDH
-56kDa
-36
C
Skov3-ip1
Hey
NC
FOXC2-sh1
FOXC2-sh1/STC1 OE
OD450
days
**
D
NC
FOXC2-sh1
FOXC2-sh1/ STC1 OE
Skov3-ip1
migration
invasion
100μm
E
Skov3-ip1
NC
FOXC2-sh1
FOXC2-sh1/STC1 OE
cell numbers/field
migration
invasion
***
**
F
NC
FOXC2-sh1
FOXC2-sh1/ STC1 OE
Hey
migration
invasion
100μm
Hey
NC
FOXC2-sh1
FOXC2-sh1/STC1 OE
cell numbers/field
migration
invasion
**
***
G
Class
WE
So
SM
PI
PE
PC
PA
LPC
DG
CerP
CerG2
Cer
Percentage difference(%)
H
Color Key
Value
Hey-NC
Hey- FOXC2-sh1
Hey- FOXC2-sh2
PC(31:1) +PE+ PI(18:0)
Cer+CerG2 +SM(d44:4)
PC(36:6) +SM(d41:4) +DG
PI(16:0) +PC(32:2) +WE
So+CerP +LPC+PC

Figure S6

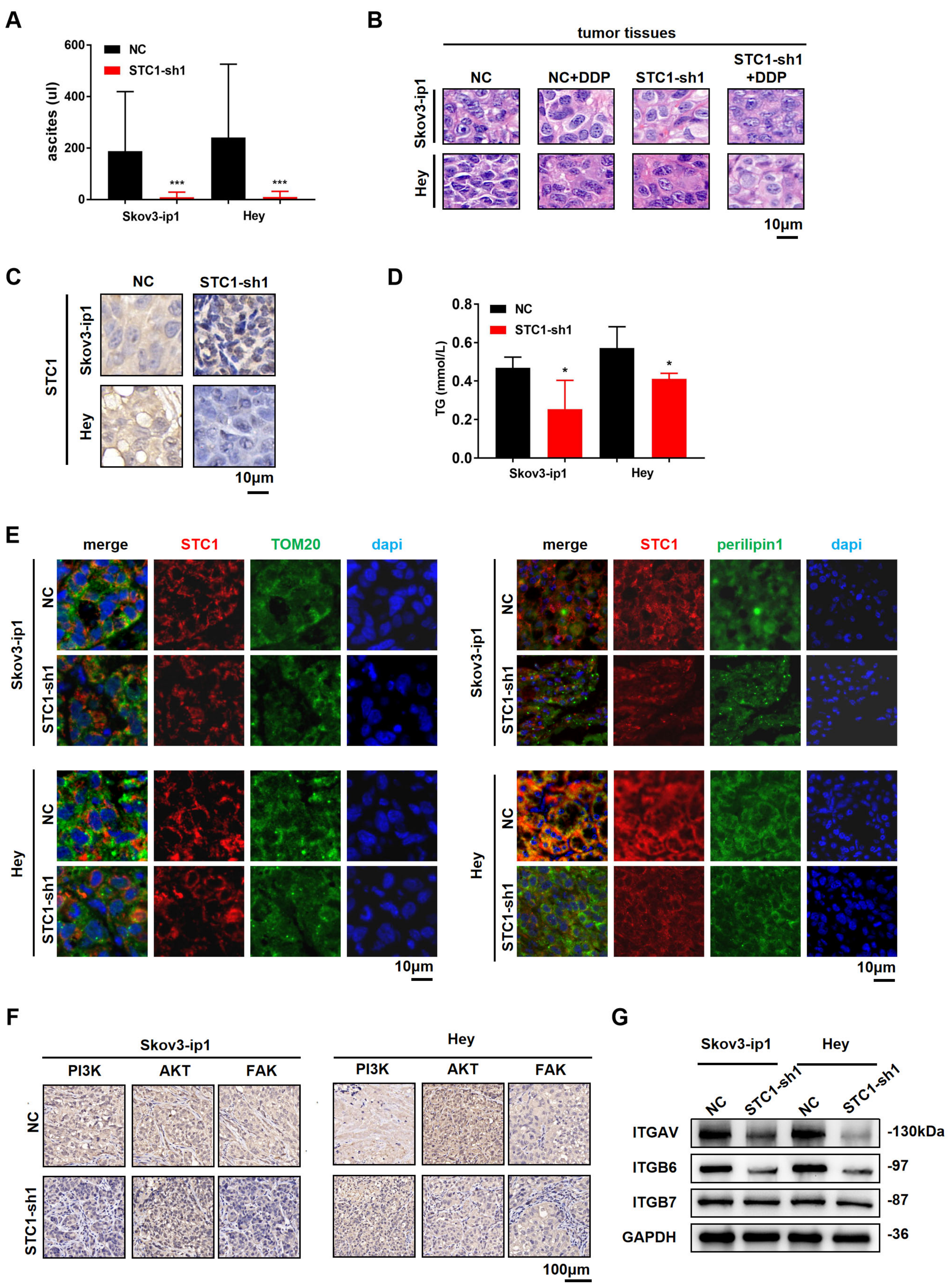

A
600
400
200
0
ascites (ul)
NC
STC1-sh1
***
***
Skov3-ip1
Hey
B
tumor tissues
NC
NC+DDP
STC1-sh1
STC1-sh1 +DDP
Skov3-ip1
Hey
10μm
C
NC
STC1-sh1
STC1
Skov3-ip1
Hey
10μm
D
0.8
0.6
0.4
0.2
0.0
TG (mmol/L)
NC
STC1-sh1
*
*
Skov3-ip1
Hey
E
merge
STC1
TOM20
dapi
perilipin1
Skov3-ip1
Hey
NC
STC1-sh1
10μm
F
Skov3-ip1
Hey
PI3K
AKT
FAK
NC
STC1-sh1
100μm
G
Skov3-ip1
Hey
NC
STC1-sh1
ITGAV
ITGB6
ITGB7
GAPDH
-130kDa
-97
-87
-36

Figure S7

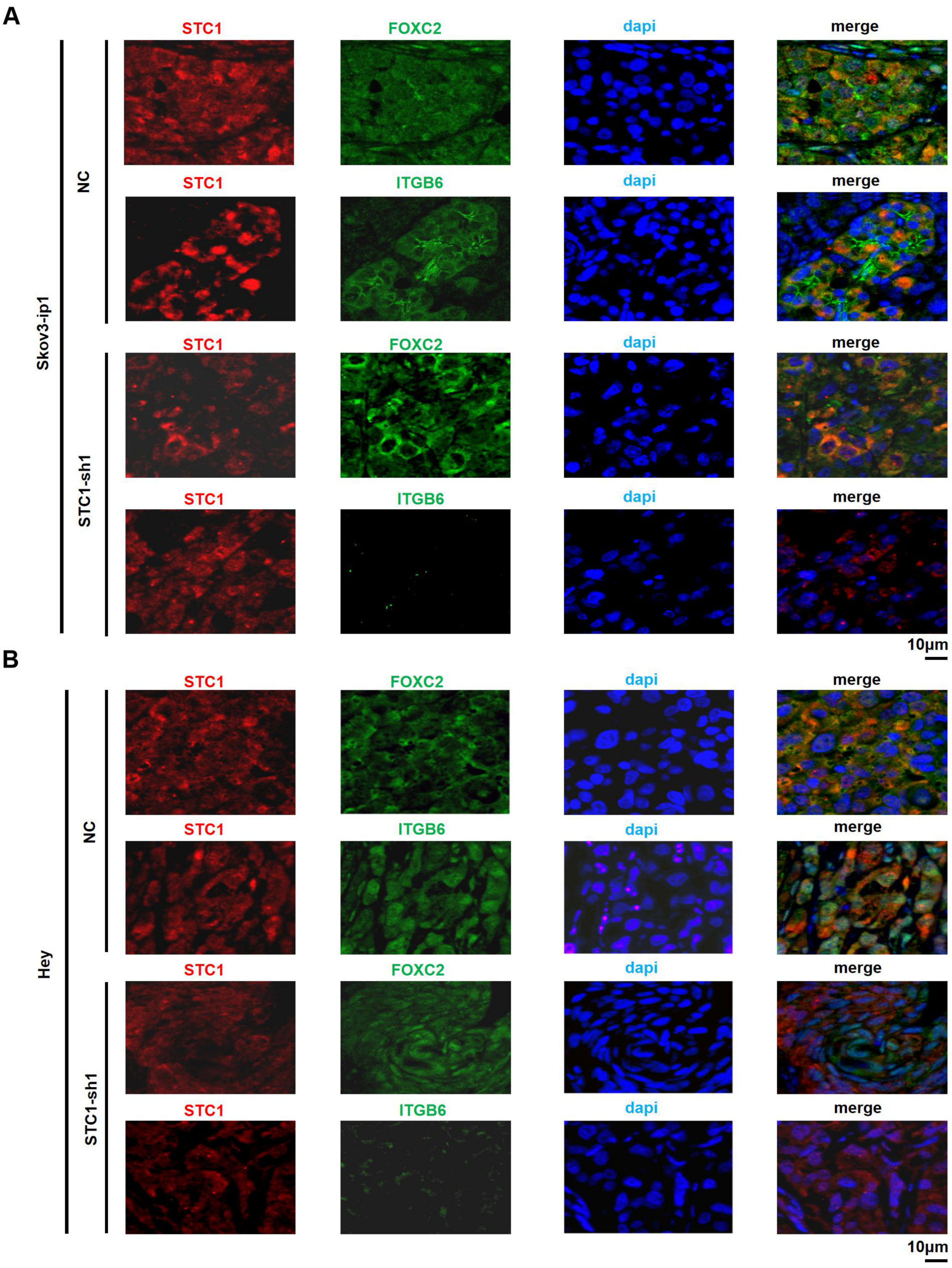
A
STC1
FOXC2
dapi
merge
STC1
ITGB6
dapi
merge
STC1
FOXC2
dapi
merge
STC1
ITGB6
dapi
merge
Skov3-ip1
NC
STC1-sh1
10μm
B
STC1
FOXC2
dapi
merge
STC1
ITGB6
dapi
merge
STC1
FOXC2
dapi
merge
STC1
ITGB6
dapi
merge
Hey
NC
STC1-sh1
10μm

Figure S8

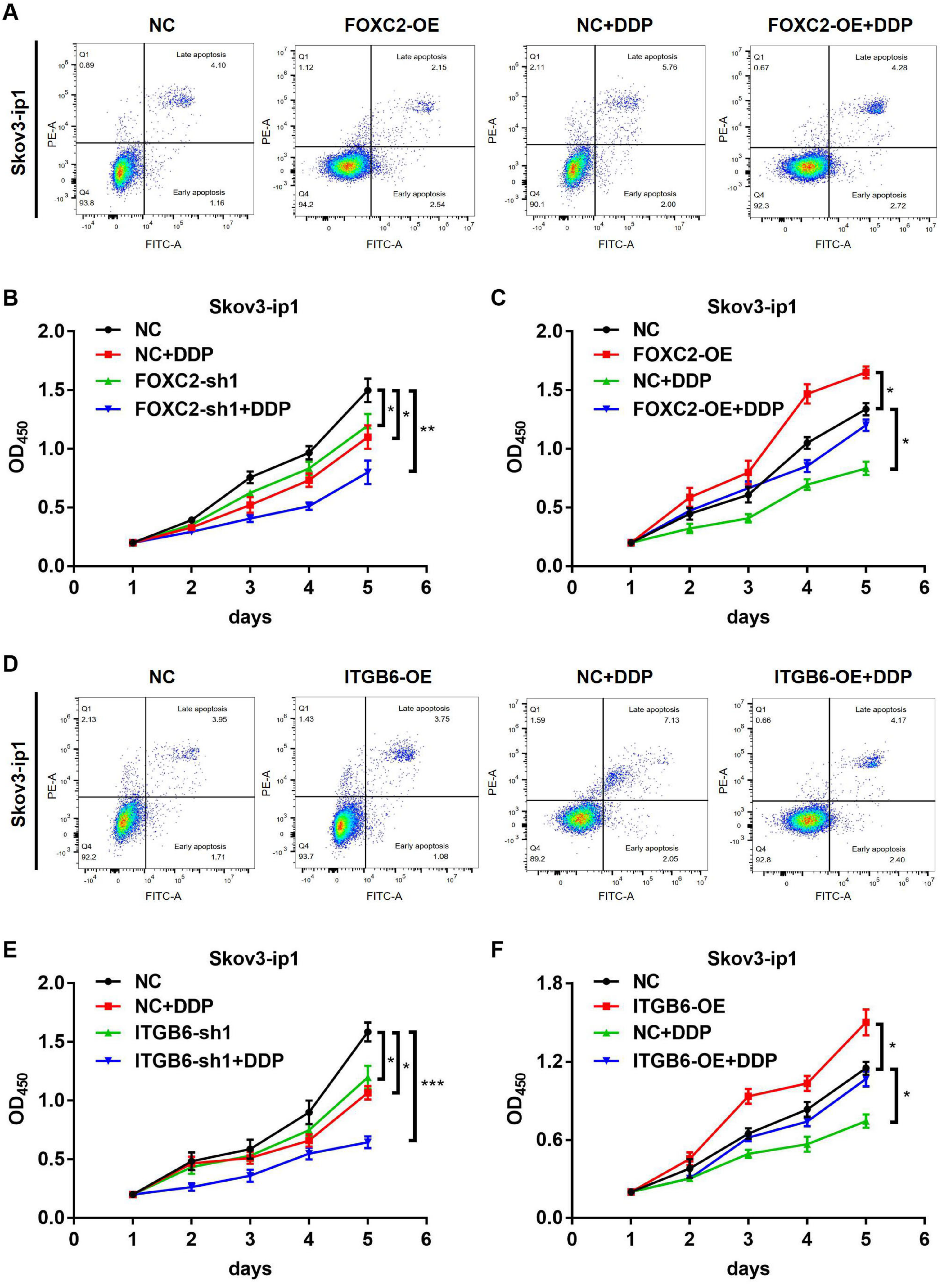

A
NC
FOXC2-OE
NC+DDP
FOXC2-OE+DDP
Skov3-ip1
Q1
0.89
Late apoptosis
4.10
Q4
93.8
Early apoptosis
1.16
Q1
1.12
Late apoptosis
2.15
Q4
94.2
Early apoptosis
2.54
Q1
2.11
Late apoptosis
5.76
Q4
90.1
Early apoptosis
2.00
Q1
0.67
Late apoptosis
4.28
Q4
92.3
Early apoptosis
2.72
PE-A
FITC-A
B
Skov3-ip1
NC
NC+DDP
FOXC2-sh1
FOXC2-sh1+DDP
OD450
days
C
Skov3-ip1
NC
FOXC2-OE
NC+DDP
FOXC2-OE+DDP
OD450
days
D
NC
ITGB6-OE
NC+DDP
ITGB6-OE+DDP
Skov3-ip1
Q1
2.13
Late apoptosis
3.95
Q4
92.2
Early apoptosis
1.71
Q1
1.43
Late apoptosis
3.75
Q4
93.7
Early apoptosis
1.08
Q1
1.59
Late apoptosis
7.13
Q4
89.2
Early apoptosis
2.05
Q1
0.66
Late apoptosis
4.17
Q4
92.8
Early apoptosis
2.40
PE-A
FITC-A
E
Skov3-ip1
NC
NC+DDP
ITGB6-sh1
ITGB6-sh1+DDP
OD450
days
F
Skov3-ip1
NC
ITGB6-OE
NC+DDP
ITGB6-OE+DDP
OD450
days

Figure S9

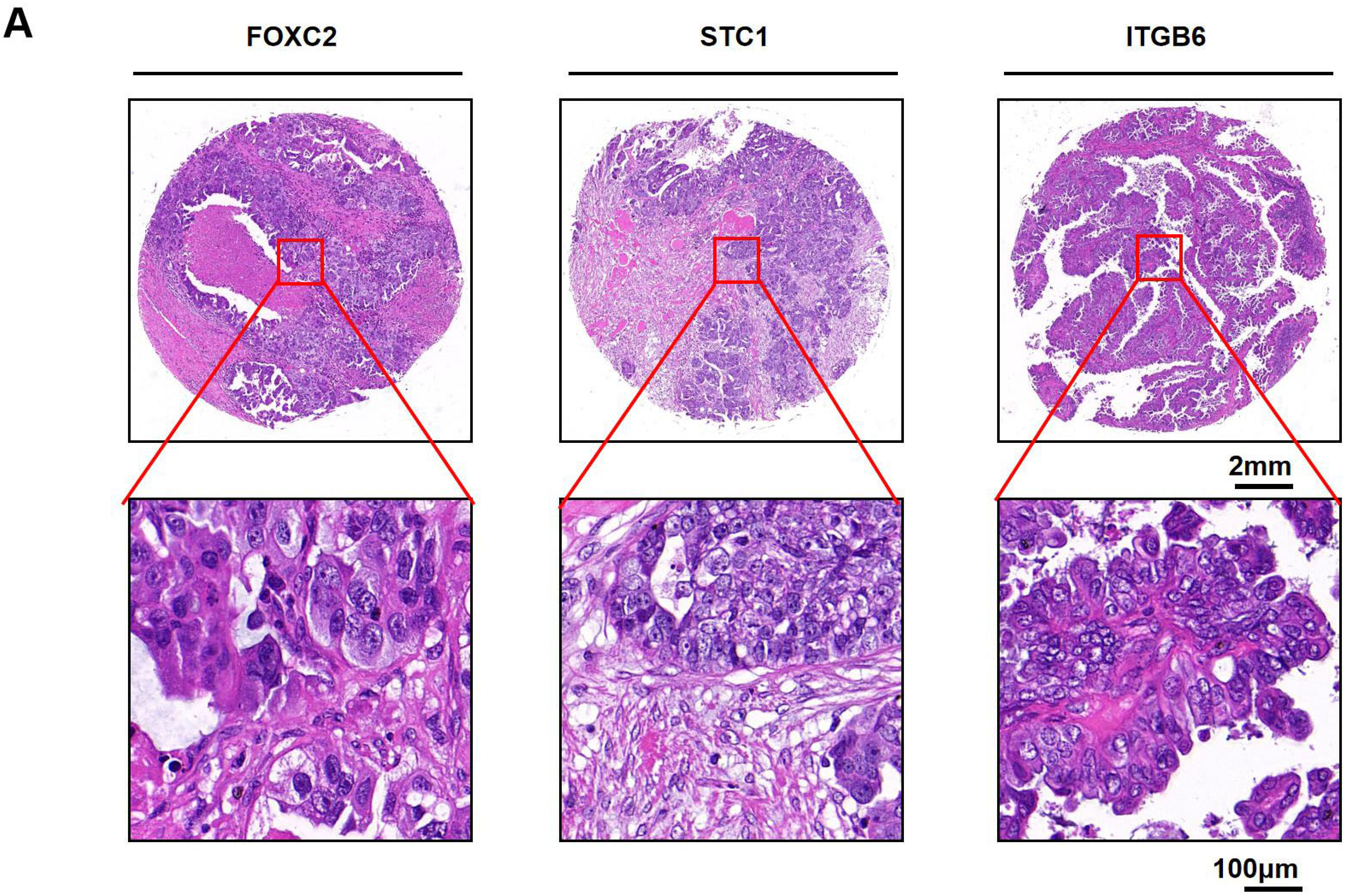

Supplementary Figure Legends

Supplementary Figure S1. STC1 is related to lipid metabolism in OC tissues. A Enrichment analysis of STC1 in different types of cell subgroups through single-cell sequencing. **B** GO analysis of tumor tissues and peritoneal metastasis tissues of OC patients. **C** GSEA of peritoneal metastasis tissues and tumor tissues of OC patients.

Supplementary Figure S2. STC1 promotes cell proliferation and cell migration *in vitro*. A Cell proliferation assays in Skov3-ip1 and Hey cell lines transfected with the NC groups, STC1-sh1 and STC1-sh2 groups. **B-C** Colony formation assays in Skov3-ip1 and Hey cell lines and statistical analysis. **D-F** Cell migration and invasion assays in Skov3-ip1 and Hey cell lines and statistical analysis. **G** Wound healing assays in Skov3-ip1 and Hey cell lines and statistical analysis. (Data are shown as the means ± SD. Significance was calculated using Student's t test. *, P < 0.05, **, P < 0.01, ***, P < 0.001, ****, P < 0.0001.)

Supplementary Figure S3. STC1 promotes lipid metabolism *in vitro*. A A total of 1412 lipid species from 30 lipid class were identified. **B** 70 significantly changed lipids from 10 lipid classes were presented. **C** IF assays detected the relationship between STC1 and TOM20 in Skov3-ip1 and Hey cell lines. **D** IF assays detected the relationship between STC1 and perilipin1 in Skov3-ip1 and Hey cell lines.

Supplementary Figure S4. ITGB6 rescues the promotion effect of STC1 *in vitro*. **A** Mass spectrometry analysis of the SDS-PAGE above. The detected peptides spectra of ITGB6 were listed. **B** The knockdown of STC1 expression level in the STC1-sh1 groups were rescued by ITGB6 OE plasmid in Skov3-ip1 and Hey cell lines. **C** Cell

proliferation assays in Skov3-ip1 and Hey cell lines in the NC groups, STC1-sh1 groups and STC1-sh1/ITGB6 OE groups. **D-F** Cell migration and invasion assays in Skov3-ip1 and Hey cell lines in the NC groups, STC1-sh1 groups and STC1-sh1/ITGB6 OE groups. **G** ORO staining in Skov3-ip1 and Hey cell lines in the NC groups, STC1-sh1 groups and STC1-sh1/ITGB6 OE groups (red arrow: TGs). **H** 11 significantly changed lipids from 5 lipid classes were identified in the ITGB6-sh1/sh2 groups compared with the NC group. **I** A heat map of significant differences in the variables among the NC group, ITGB6-sh1 and ITGB6-sh2 groups of Hey cells. The variables were ranked by ANOVA. (Data are shown as the means ± SD. Significance was calculated using Student's t test. *, P < 0.05, **, P < 0.01, ***, P < 0.001, ****, P < 0.0001.)

Supplementary Figure S5. STC1 rescues the promotion effect of FOXC2 *in vitro*. **A** FOXC2 has interaction with STC1 ($p=4.909e^{-5}$). **B** The knockdown of FOXC2 expression level in the FOXC2-sh1 groups were rescued by STC1 OE plasmid in Skov3-ip1 and Hey cell lines. **C** Cell proliferation assays in Skov3-ip1 and Hey cell lines in the NC groups, FOXC2-sh1 groups and FOXC2-sh1/STC1 OE groups. **D-F** Cell migration and invasion in Skov3-ip1 and Hey cell lines in the NC groups, FOXC2-sh1 groups and FOXC2-sh1/STC1 OE groups. **G** 87 significantly changed lipids from 12 lipid classes were identified in the FOXC2-sh1/sh2 groups compared with the NC group. **H** A heat map of significant differences in the variables among the NC group, FOXC2-sh1 groups and FOXC2-sh2 groups of Hey cells. The variables were ranked by ANOVA. (Data are shown as the means ± SD. Significance was

calculated using Student's t test. *, $P < 0.05$, **, $P < 0.01$, ***, $P < 0.001$, ****, $P < 0.0001$.)

Supplementary Figure S6. STC1 promotes metastasis and lipid metabolism *in vivo*. A The NC groups showed significantly more ascites than the STC1-sh1 groups. **B** H&E staining of tumor tissues in four groups. **C** IHC assays of STC1 in OC tissues. **D** The NC groups showed higher level of blood TG compared with the STC1-sh1 groups. **E** IF assay detected the relationship between STC1, TOM20 and perilipin1 in two cell lines in mouse tumor tissues. **F** IHC assays of proteins in PI3K signaling pathway in mouse tumor tissues. **G** STC1 influenced the protein expression levels of members in the integrin family in mouse tumor tissues by WB assays. (Data are shown as the means ± SD. Significance was calculated using Student's t test. *, $P < 0.05$, **, $P < 0.01$, ***, $P < 0.001$, ****, $P < 0.0001$.)

Supplementary Figure S7. The expression of FOXC2, STC1 and ITGB6 in mouse tumor tissues. A-B IF assay detected the relationship of STC1 and FOXC2, STC1 and ITGB6 in mouse tumor tissues in two cell lines.

Supplementary Figure S8. Targeting the FOXC2/ITGB6 signaling axis is related to DDP chemoresistance *in vitro*. A Cell apoptosis results in the NC, FOXC2-OE, NC+DDP and FOXC2-OE+DDP groups in Skov3-ip1 cell lines. **B** The results of cell proliferation assays in the NC, NC+DDP, FOXC2-sh1 and FOXC2-sh1+DDP groups in Skov3-ip1 cells. **C** The results of cell proliferation assays in the NC, FOXC2-OE, NC+DDP and FOXC2-OE+DDP groups in Skov3-ip1 cell lines. **D** Cell apoptosis results in the NC, ITGB6-OE, NC+DDP and ITGB6-OE+DDP groups in Skov3-ip1

cell lines. **E** The results of cell proliferation assays in the NC, NC+DDP, ITGB6-sh1 and ITGB6-sh1+DDP groups in Skov3-ip1 cells. **F** The results of cell proliferation assays in the NC, ITGB6-OE, NC+DDP and ITGB6-OE+DDP groups in Skov3-ip1 cell lines. (Data are shown as the means ± SD. Significance was calculated using Student's t test. *, $P < 0.05$, **, $P < 0.01$, ***, $P < 0.001$, ****, $P < 0.0001$.)

Supplementary Figure S9. H&E staining of the TMAs of FOXC2, STC1 and ITGB6 in OC tissues. A H&E staining of the TMAs of FOXC2, STC1 and ITGB6 in OC tissues.
